# A Novel Y-Specific Long Non-Coding RNA Associated with Cellular Lipid Accumulation in HepG2 cells and Atherosclerosis-related Genes

**DOI:** 10.1038/s41598-017-17165-9

**Published:** 2017-12-01

**Authors:** Elsa Molina, Guat S. Chew, Stephen A. Myers, Elyse M. Clarence, James M. Eales, Maciej Tomaszewski, Fadi J. Charchar

**Affiliations:** 1 School of Applied and Biomedical Sciences, Faculty of Science and Technology, Federation University, Mount Helen Campus, Ballarat, VIC Australia; 2 School of Health Sciences, Faculty of Health, University of Tasmania, Newnham Campus, Launceston, TAS Australia; 3 Institute of Cardiovascular Sciences, The University of Manchester, Manchester, UK; 4 Department of Physiology, University of Melbourne, Melbourne, Australia; 5 Department of Cardiovascular Sciences, University of Leicester, Leicester, UK

## Abstract

There is an increasing appreciation for the role of the human Y chromosome in phenotypic differences between the sexes in health and disease. Previous studies have shown that genetic variation within the Y chromosome is associated with cholesterol levels, which is an established risk factor for atherosclerosis, the underlying cause of coronary artery disease (CAD), a major cause of morbidity and mortality worldwide. However, the exact mechanism and potential genes implicated are still unidentified. To date, Y chromosome-linked long non-coding RNAs (lncRNAs) are poorly characterized and the potential link between these new regulatory RNA molecules and hepatic function in men has not been investigated. Advanced technologies of lncRNA subcellular localization and silencing were used to identify a novel intergenic Y-linked lncRNA, named lnc-KDM5D-4, and investigate its role in fatty liver-associated atherosclerosis. We found that lnc-KDM5D-4 is retained within the nucleus in hepatocytes. Its knockdown leads to changes in genes leading to increased lipid droplets formation in hepatocytes resulting in a downstream effect contributing to the chronic inflammatory process that underpin CAD. Our findings provide the first evidence for the implication of lnc-KDM5D-4 in key processes related to fatty liver and cellular inflammation associated with atherosclerosis and CAD in men.

## Introduction

The human Y chromosome is best known for its key role in spermatogenesis and testicular development^1^. It is becoming increasingly clear that the biological functions of the Y chromosome transcend the male fertility and reproduction^2^, with associations between genetic variation within the male-specific region of the Y chromosome (MSY) and complex polygenic phenotypes including blood pressure^3^, circulating cholesterol levels^4^, and coronary artery disease (CAD)^2,5^ being reported. The mechanisms of these associations are not entirely clear although it appears that they are (at least in part) mediated by the expression of MSY genes in the target cells and tissues^6^. To date, over 200 Y-linked genes have been identified and those that encode Y proteins have been well-characterised^7^. However, MSY harbours not only 27 protein-coding genes but also a number of long non-coding RNAs (lncRNAs) – important new regulatory molecules implicated in cardiovascular health^8^ and disease^9^. So far, there is no data on Y chromosome-linked lncRNAs in relation to any human phenotypes. To this end, we sought to investigate Y-linked long intergenic non-coding RNAs (lincRNAs) - the largest class within the lncRNA group^10^ - in relation to phenotypes of relevance to CAD such as fatty liver-associated atherosclerosis.

Here, we studied for the first time eleven novel Y-linked lincRNAs by real-time PCR gene expression analysis in the human hepatoma HepG2 male cell line, an *in vitro* model system used in previous studies on liver steatosis and CAD^11–14^. We also investigated the expression of a novel intergenic Y-linked lincRNA, named lnc-KDM5D-4, across 21 normal tissue types. Utilising advanced technologies of lncRNA subcellular localization and silencing, we further characterized lnc-KDM5D-4 within HepG2 cells, and investigated the effects of its knockdown on atherosclerosis-related genes in these cells. Throughout the text, ‘lnc-KDM5D-4′ refers to the long non-coding RNA gene, and ‘lnc-KDM5D-4:1′ refers to the long non-coding RNA transcript.

## Materials and Methods

### Primer design

The human lincRNA catalogues^15,16^ were used to select Y-specific lincRNA genes based on their expression in tissues relevant to the liver and CAD, such as heart, liver and white blood cells (WBCs) (Supplementary Materials and Methods). Primers for SYBR® Green-based real-time polymerase chain reaction (PCR) gene expression analysis were designed using the free online software Primer-BLAST (Basic Local Alignment Search Tool) following the recommendations for probe melting temperature (Tm) values, GC content, and amplicon length provided by Thornton & Basu^17^. All the primers for lincRNAs were designed from the DNA template sequence annotated on *LNCipedia*, the database for human lncRNA genes and transcripts^18^. Primer sequences are available in Supplementary Table S1.

### HepG2 cell culture and treatments

The human hepatoma cell line, HepG2, obtained by the Charchar Laboratory, was grown as a monolayer culture in Dulbecco’s modified Eagle medium (DMEM, 1X) (Gibco^®^, Life Technologies^™^) supplemented with 10% (v/v) heat inactivated foetal bovine serum (HI-FBS) (Gibco^®^, Life Technologies^™^), 100 U/mL penicillin, 100 µg/mL streptomycin (Gibco^®^, Life Technologies^™^) and were incubated at 37 °C in a humidified atmosphere containing 5% (v/v) CO_2_ in the air. The HepG2 cell model of palmitate-induced steatosis and insulin-resistance were developed as previously described^19^ (Supplementary Materials and Methods).

### Human tissue RNA panel

The Human Tissue RNA Panel was obtained from Ambion^®^ FirstChoice^®^Human. Total RNA Survey Panel which consists of 10 µg pools of total RNA (stored in 1 mmol/L of sodium citrate) from 20 different normal, human tissues including adipose, bladder, brain, cervix, colon, esophagus, heart, kidney, liver, lung, ovary, placenta, prostate, skeletal muscle, small intestine, spleen, testes, thymus, thyroid, and trachea. Each pool is comprised of RNA from 3 tissue donors (with at least 1 male donor per tissue except for the cervix, placenta, and ovary RNA sample pools) and underwent a stringent DNase treatment. After storage at −80 °C on arrival, RNA samples were thawed at 37 °C then placed on ice prior use as recommended by the manufacturer’s instructions.

### Blood collection

To study the expression of lincRNAs expressed in leucocytes in men, five healthy male volunteers (positive control group) and two healthy females (negative control group), between 18–50 years old were recruited at the Federation University Australia (Mount Helen campus). Participants were required to meet the following inclusion criteria: current non-smokers, free of recent surgery, ambulatory aids, and acute joint injury, free of underlying medical conditions such as heart disease, un-medicated hypertension and respiratory disorders in order to avoid skewing the results. Participants’ blood was withdrawn in Ethylenediaminetetraacetic acid (EDTA) blood collection tubes (tubes commonly used in routine haematology and furnished by the phlebotomist) by a qualified phlebotomist at Mount Helen campus. Then, blood samples were immediately stored on ice and total RNA was purified within an hour of collection to preserve the RNA integrity. This study was approved by the Federation University Ethics Committee at Mount Helen campus and carried out in accordance with Federation University relevant guideline and regulations. All the subjects gave informed consent [Ethics Project Number: B13-020].

### Oil Red O cell staining

To confirm steatosis induced by palmitate in HepG2 cells, cellular lipid droplets were stained using Oil Red O (ORO) from Hepatic Lipid Accumulation/Steatosis Assay (abcam^®^) (Supplementary Materials and Methods).

### Protein extraction and Western Blot analysis

Total cellular protein from HepG2 cells treated with insulin over 48 h was isolated by scraping the cells with Pierce® RIPA buffer. A total soluble protein (50 μg) was resolved on a 4–15% SDS-PAGE gradient gel (BIORAD®) and transferred to a nitrocellulose membrane (Supplementary Materials and Methods).

### Total RNA isolation

Total RNA was extracted from cells using TRIzol reagent (Ambion^®^). Total RNA was resuspended in 50 µL RNase-free water followed by a column DNase treatment (1 µL per RNA sample) using Recombinant DNase I (Ambion^®^, Applied Biosystem^®^) according to the manufacturer’s instructions. RNA yield and purity were measured by absorbance using a NanoDrop^™^ 2000 Spectrophotometer (Thermo Fisher Scientific^®^, Australia). The ratio (A_260 nm_/A_280 nm_) of ~2.0 was accepted as “pure” for RNA. Finally, RNA samples were stored at −80 °C until use.

### Synthesis of cDNA and real-time PCR

RNA (2 µg) was reverse transcribed into cDNA with the High Capacity Reverse Transcription Kit (Life Technologies^®^). Real-time PCR was assessed in triplicate and carried out using SYBR^®^ Green (Bioline^®^) according to the manufacturer’s instructions. Data were analysed using the 2^-delta delta CT^ method^20^. C_T_ results were normalised with two housekeeping genes, *ACTB* (or *GAPDH*) and *UBC* (Supplementary Materials and Methods).

### RNA fluorescence *in situ* hybridization (RNA FISH)

RNA FISH was performed using the advanced QuantiGene^®^ ViewRNA ISH Cell Assay (Affymetrix^®^). The permeabilisation procedure was amended and 1% glacial acetic acid was added to the fixation solution to detect nuclear RNAs^21^, then the RNA FISH assay from Four-Chambered Dish Format protocol was performed according to the manufacturer’s instructions (Supplementary Materials and Methods).

### Nuclear and Cytoplasmic RNA fraction isolations

Nuclear and cytoplasmic RNA fractions were isolated separately from confluent cells using the Cytoplasmic & Nuclear RNA Purification Assay (Norgen Biotek Corp.^®^, Canada) according to the manufacturer’s instructions. Both RNA fractions, nuclear and cytoplasmic, were resuspended in 50 µL RNase-free water followed by a column DNase treatment (1 µL per 1000 ng of RNA) using the Recombinant DNase I (Ambion^®^, Applied Biosystem^®^) according to manufacturer’s instructions. RNA yield and purity were measured by absorbance using a NanoDrop^™^ 2000 Spectrophotometer (Thermo Fisher Scientific^®^, Australia). The ratio (A_260 nm_/A_280 nm_) of ~2.0 was accepted as “pure” for RNA. Finally, the RNA samples were stored at −80 °C until use.

### Cell transfection and lncRNA knockdown

Advanced antisense oligonucleotides (AOs) Locked Nucleic Acid (LNA^™^) longRNA GapmeRs - a class of high-affinity RNA analogues that exhibit high thermal stability when hybridized to a complementary DNA or RNA strand - were used to silence lnc-KDM5D-4. Antisense LNA^™^ longRNA GapmeRs were custom designed (except for the control GapmeR ‘Negative control A’) and synthesized by Exiqon^®^. Three different GapmeRs targeting three different sites of lnc-KDM5D-4:1 RNA were used following the Exiqon’s recommendations. The transfection of the following GapmeRs, lnc-KDM5D-4_GapmeR_1 (Product sequence 5′-3′: GAGATGAAGCGGAATT), lnc-KDM5D-4_GapmeR_2 (Product sequence 5′-3′: CTTACTTTAGACTTC), lnc-KDM5D-4_GapmeR_3 (Product sequence 5′-3′: ACGTAAAATAGGATTA), and Negative control A (Product sequence 5′-3′: AACACGTCTATACGC) were performed using Lipofectamine^®^ 2000 (Thermo Fisher Scientific^®^) following Exiqon manufacturer’s recommendations as well as those for knockdown of lncRNA and AOs provided by Integrated DNA Technologies^®^’s webinars on www.youtube.com/user/idtdnabio. HepG2 cells were seeded into 6-well plates at a density of ~2 × 10^5^/mL and cultured in DMEM supplemented with 10% HI-FBS, penicillin (100 U/mL), and streptomycin (100 µg/mL) until the cells reached ~70% confluence. Then, the cells were transfected with 10 nmol/L of AO in Lipofectamine^®^ 2000 reagent. After 48 h, total RNA was extracted using TRIzol reagent followed by a RNA clean-up step using miRNeasy kit (Qiagen^®^). Then, the knockdown was confirmed by real-time PCR.

### Reverse transcription and Atherosclerosis RT^2^ Profiler™ PCR Array

The Human Atherosclerosis RT^2^ Profiler PCR Array (PAHS-038ZE) from SA Biosciences (Qiagen^®^; Catalogue No. 330231) was performed following the manufacturer’s recommendations (Supplementary Materials and Methods). Then, the human tissue-specific network webserver GIANT^22^ was used to generate the potential tissue-specific functional interactions between the atherosclerosis-relevant genes.

### Statistical analysis

Statistical analysis of data was performed using Prism (GraphPad Software). Data were analysed using a Student’s unpaired *t*-test to test significance of data comparisons between cell treatments, with *p*-values < 0.05 considered as statistically significant.

## Results

### A survey of the expression of novel Y chromosome-linked lincRNAs in HepG2 cells

**Figure 1:**
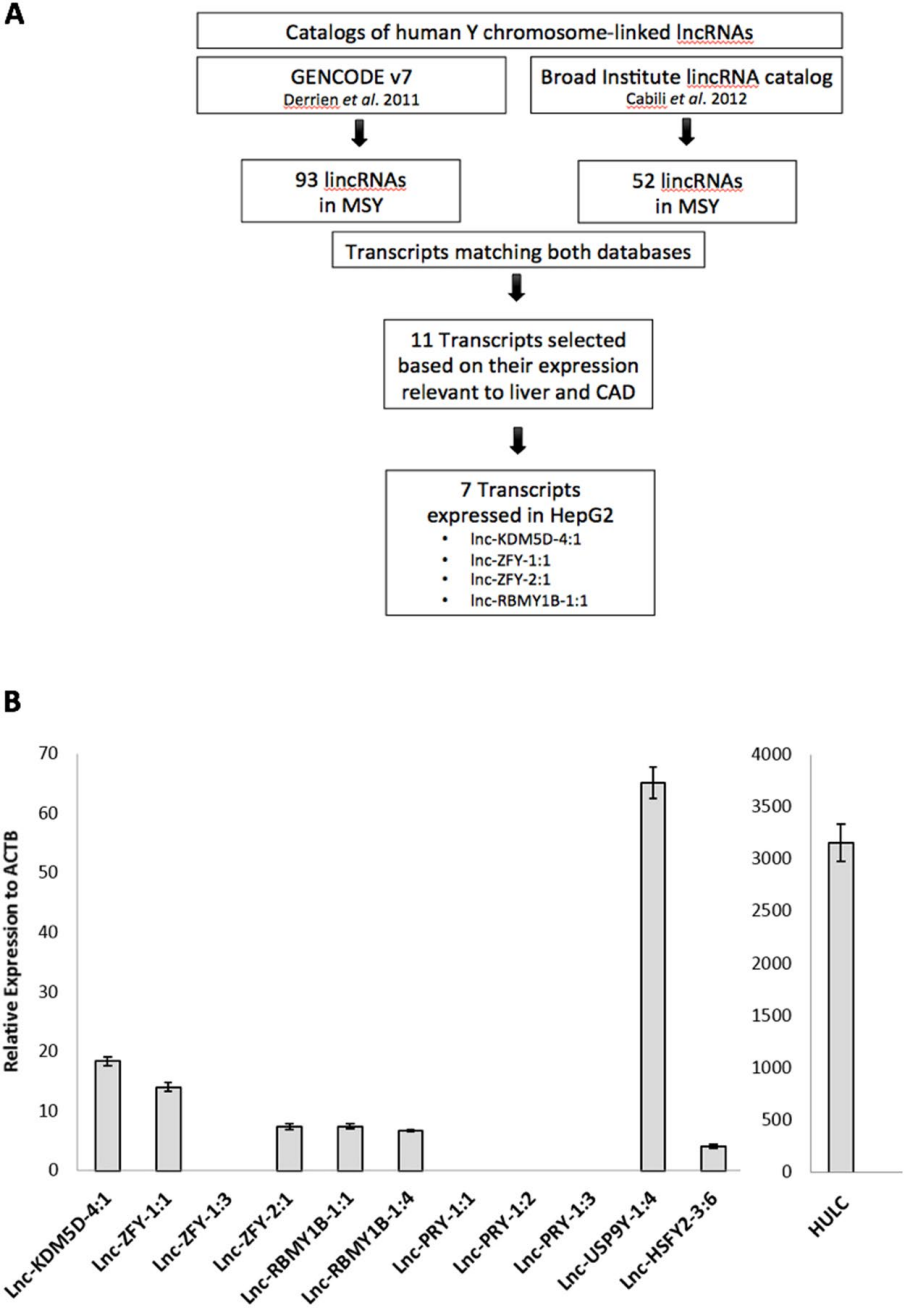
Identification of Y chromosome lincRNAs in HepG2 cells. (**A**) Steps followed in the identification process of Y-specific lincRNAs. (**B**) Y-specific lincRNA real-time PCR analysis. Lnc-KDM5D-4:1, lnc-ZFY-1:1, lnc-ZFY-1:3, lnc-ZFY-2:1, lnc-RBMY1B-1:1, lnc-RBMY1B-1:4, lnc-PRY-1:1, lnc-PRY-1:2, lnc-PRY-1:3, lnc-USP9Y-1:4, and lnc-HSFY2-3:6 normalised expression values with respective S.E.M. relative to actin beta (ACTB) housekeeping gene. HULC – a lincRNA with confirmed up-regulation in HepG2 cells - used as a positive control (*n* = 3).

Using human lncRNA reference catalogues^15,16^ which include the Y chromosome, we identified 11 candidate MSY lincRNA transcripts and measured their expression in the human hepatoma HepG2 cell line. Seven MSY lincRNA transcripts such as lnc-KDM5D-4:1, lnc-ZFY-1:1, lnc-ZFY-2:1, lnc-RBMY1B-1:1, lnc-RBMY1B-1:4, lnc-USP9Y-1:4, and lnc-HSFY2-3:6 were found to be expressed in the HepG2 cells (Fig. 1A,B). Their average abundance was significantly lower than that of lncRNA HULC (Hepatocellular carcinoma up-regulated long non-coding RNA) which was used as a positive control^23^.

### The effects of fatty acid palmitate on the transcript expression of Y chromosome-linked lincRNAs in a steatosis HepG2 cell line

**Figure 2:**
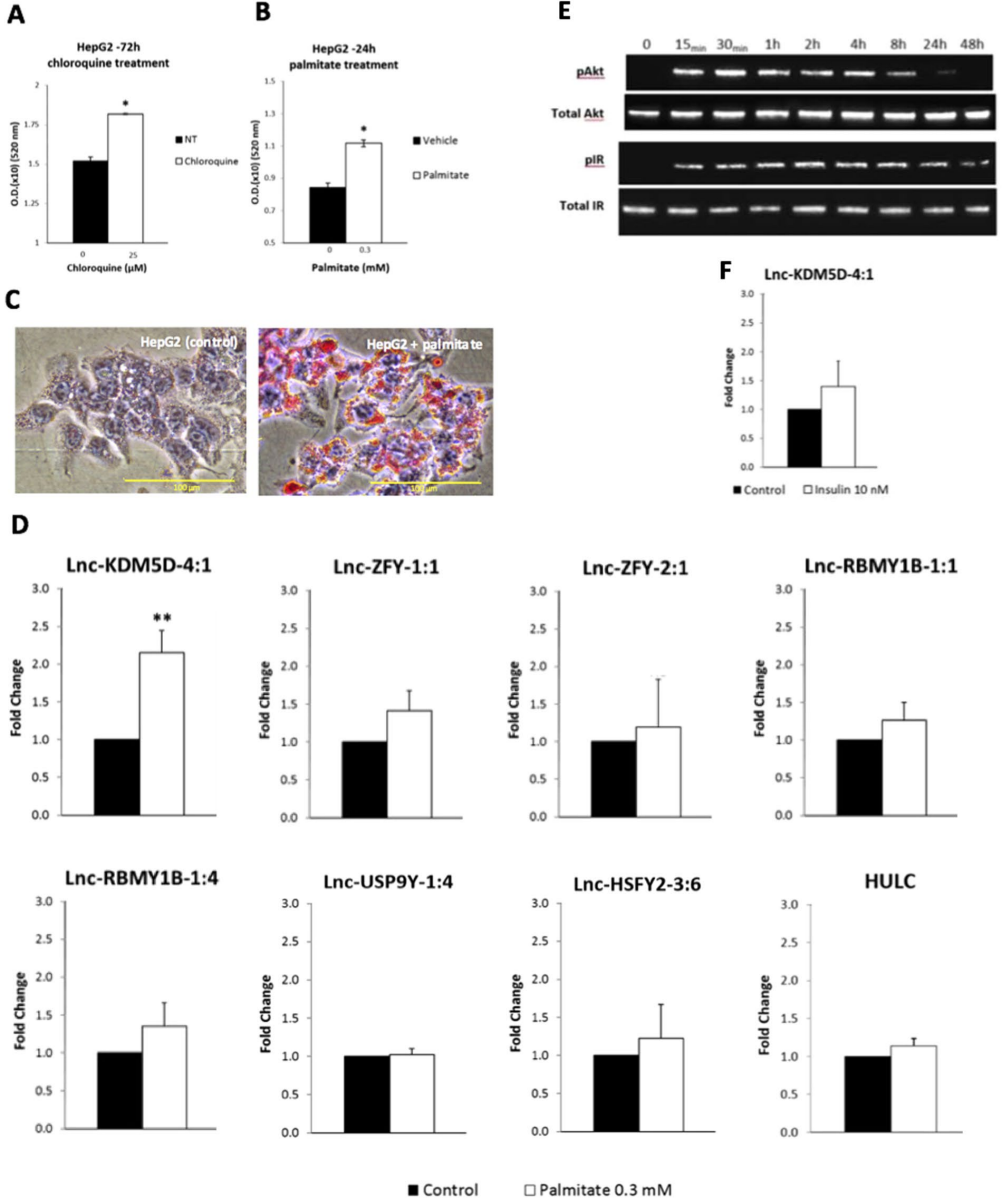
Expression of MSY lincRNAs in palmitate-induced steatosis and in insulin-resistant HepG2 cells. (**A**,**B**) Quantification of the lipid accumulation by absorbance measurement after treatment with palmitate at 0.3 mmol/L (0.3 mM) (**A**), and chloroquine (as positive control) at 25 µmol/L (25 µM) (**B**). Microscopy results of the intracellular distribution of lipid droplets stained in red by the Oil Red O solution after 24 h of treatment with either the vehicle or with the palmitate (**C**). The addition of palmitate after 24 h triggers a significant increase (Fold Change = 2.16; *p*-value = 0.00216) in the expression of lnc-KDM5D-4:1 in HepG2 cells in comparison to cells not treated with palmitate (control cells) (**D**). No significant increase in the expression of lnc-ZFY-1:1, lnc-ZFY-2.1, lnc-RBMY1B-1:1, lnc-RBMY1B-1:4, lnc-USP9Y-1:4, lnc-HSFY2-3:6, and HULC is observed. (**E**) Western blot analysis of phosphorylated protein kinase B (pAkt), Total Akt, phosphorylated insulin-receptor (pIR), and Total IR (gel cropped). Antibodies were used to immunodetect the levels of pAkt, Total Akt, pIR and Total IR in an increasingly insulin-resistance HepG2 cell line. For protein phosphorylation detection, 10 nM insulin was added for 30 min before cell lysates harvest at the indicated time. Western blot analysis was performed on two independent experiments (Uncropped gel images Supplementary Figure S1). (**F**) Real-time PCR results of the 24 h insulin-stimulated HepG2 cells with no significant changes in the expression of lnc-KDM5D-4:1 in comparison to the cells not treated (control cells) (Fold Change = 1.39; *p*-value > 0.05). Data are relative to the housekeeping gene actin beta (ACTB). Error bars indicate ± SD calculated from sextuplicate wells (**A**,**B**). NT, no treatment; (*n* = 4). Statistical significance: *p-value < 0.05; **p-value < 0.01.

Positive correlations between CAD and high levels of free fatty acids (FFAs) in steatosis hepatocytes have been previously demonstrated *in vitro* using HepG2 cells^11–14^. To create a FFA-induced steatosis liver cell line, HepG2 cells were stimulated with 0.3 mmol/L of palmitate for 24 hours to induce lipid accumulation within the cells^14^. Two methods were used to assess the results: quantification of the lipid accumulation by absorbance measurement (Fig. 2A,B) and examination of the intracellular distribution of lipid droplets by Oil Red O (ORO) staining using microscopy (Fig. 2C). To confirm the ability of our assay to generate the required cell phenotypes, we compared the magnitude of lipid accumulation within the target cells with that generated by the positive control for inducing steatosis, the chloroquine (based on manufacturer’s recommendations). Results with chloroquine showed a similar significant increase in lipid accumulation within the treated cells when compared to the control cells (Fig. 2A). The absorbance measurement of the lipid quantification (Fig. 2B) suggested that HepG2 cells had acquired the palmitate-induced steatosis phenotype. These results were further validated by microscopy demonstrating a clear increase of ORO-stained lipid droplets (red) in the peri-nuclear region of the cells in comparison to the control cells (Fig. 2C). Real-time PCR results showed that 24 h-treatment with palmitate triggered a significant 2.16-fold increase in the expression of lnc-KDM5D-4:1 (*p*-value = 0.00216) in HepG2 cells. No significant increase in the expression of the transcripts lnc-ZFY-1:1, lnc-ZFY-2:1, lnc-RBMY1B-1:1, lnc-RBMY1B-1:4, lnc-USP9Y-1:4, lnc-HSFY2-3:6, and HULC was observed (*p*-value > 0.05) (Fig. 2D).

### The effects of insulin on the lncRNA transcript lnc-KDM5D-4:1 expression in an insulin-resistant HepG2 cell line

To investigate the possible correlation between steatosis and insulin-resistance, the potential effect of insulin resistance on the expression of lnc-KDM5D-4 was studied. To create an insulin-resistant cell line, HepG2 cells were stimulated with 10 nM of insulin solution over 0, 0.25, 0.5, 2, 4, 8, 24, and 48 h replacing the media and insulin every 8 h. Total cellular protein was extracted using RIPA buffer and 20 μg of total cellular protein was subjected to SDS-PAGE, and subsequently blotted onto Immobilon-PVDF membrane. The relative levels of phosphorylated protein kinase B (pAkt) or phosphorylated insulin-receptor (pIR) were detected relative to Total Akt and Total IR respectively (Fig. 2E). Western blot analysis showed that following 30 min of insulin treatment, there was an increase in the levels of pAkt that were further increased at 2 and 4 h of insulin treatment, as similarly for pIR. The level of phosphorylation then rapidly decreased at 8 h and became almost undetectable at 24 and 48 h. The level of phosphorylation of the IR decreased from 24 h. Overall, there was no change in Total Akt and IR proteins observed, suggesting that the cells had obtained the insulin-resultant phenotype (Fig. 2E). To determine if lnc-KDM5D-4 responded to insulin-resistance, its expression was analysed by real-time PCR. Results showed no significant change in the expression of lnc-KDM5D-4 in cells stimulated with insulin for 24 h compared to the control cells (Fig. 2F).

### A survey of the expression of the lncRNA transcript lnc-KDM5D-4:1 in 21 different normal human tissues

**Figure 3:**
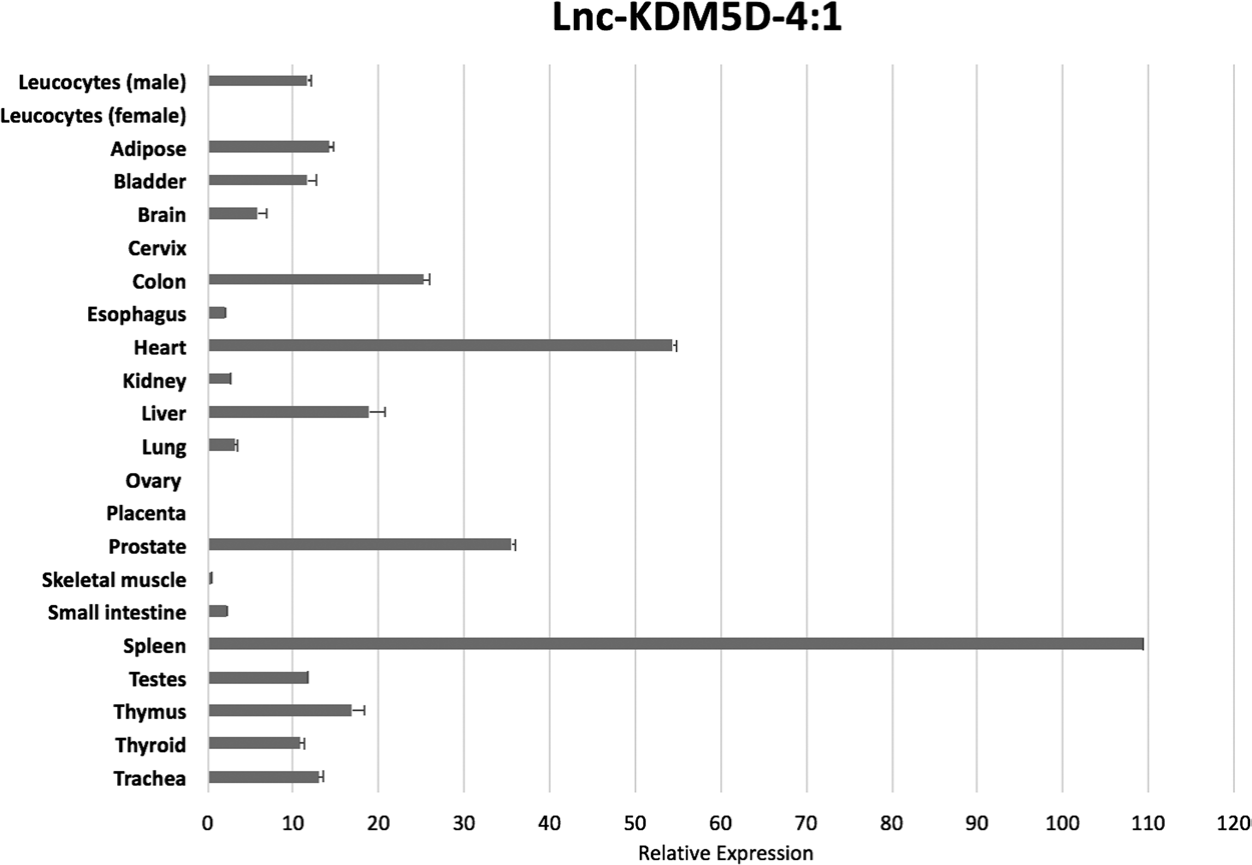
Lnc-KDM5D-4 gene expression analysis in healthy human tissues. The expression of lnc-KDM5D-4:1 in 21 different normal, human tissues – real-time PCR (no expression observed in female tissues such as cervix, ovary, placenta, and female leucocytes), the expression estimates are relative to glyceraldehyde-3-phosphate dehydrogenase (GAPDH), and error bars indicate ± S.E.M. (*n* = 3).

To gain a better understanding about the biological role of lnc-KDM5D-4, we examined its expression across 21 normal human tissue types including adipose, bladder, brain, cervix, colon, esophagus, heart, kidney, liver, lung, ovary, placenta, prostate, skeletal muscle, small intestine, spleen, testes, thymus, thyroid, trachea, male and female leucocytes from WBCs. The real-time PCR analysis revealed that lnc-KDM5D-4 shows ubiquitous expression with the highest level of expression in the spleen and the heart. As expected, no expression was found in female tissues such as cervix, ovary, and placenta as well as in female leucocytes (Fig. 3).

### Subcellular localization of lnc-KDM5D-4:1 transcripts in HepG2 cells

**Figure 4:**
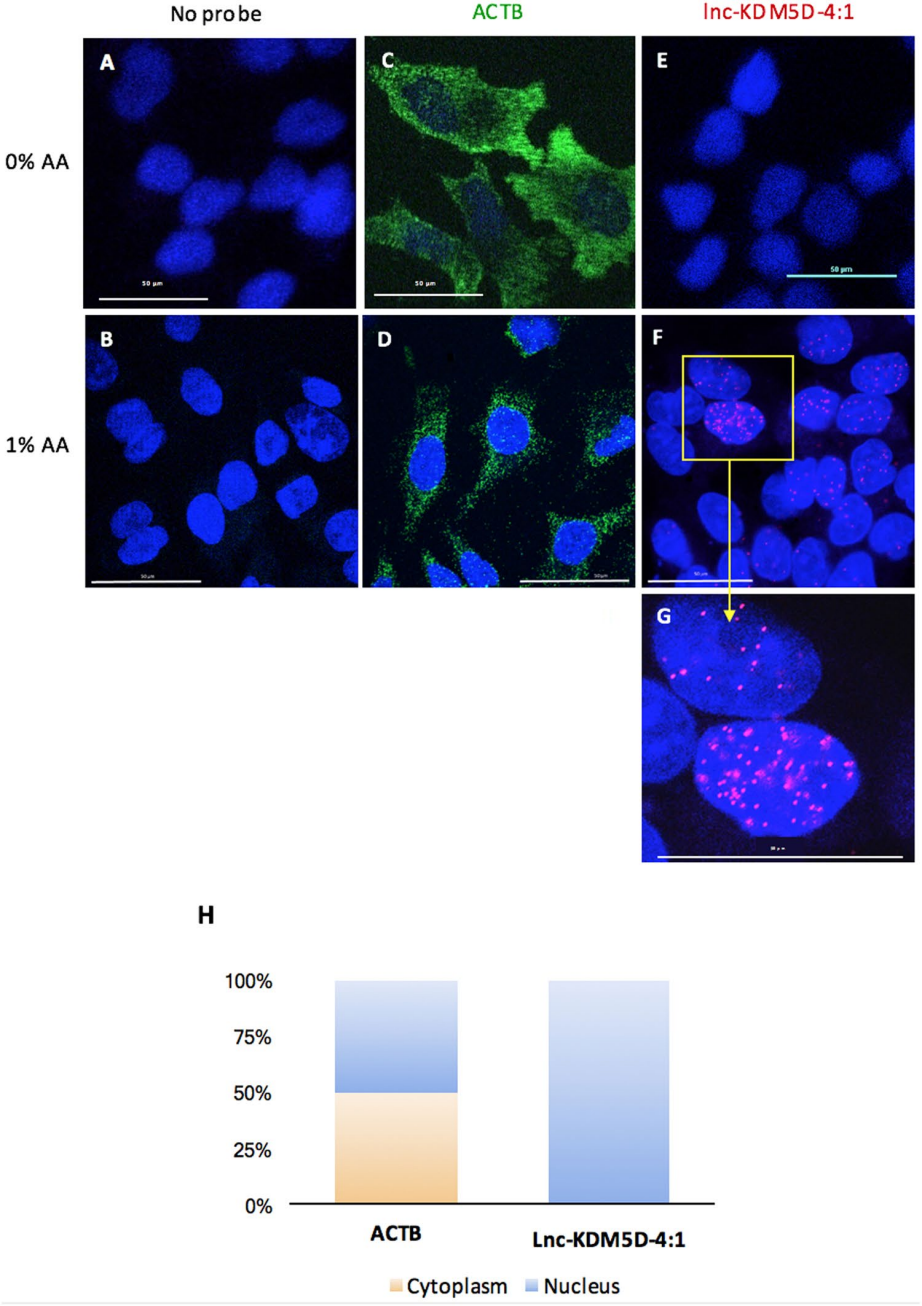
Subcellular localization of lnc-KDM5D-4:1 in HepG2 cells. (**A,B**) Cells without probes (negative control). (**C**,**D**) actin beta (ACTB fluorescence *in situ* hybridization (ACTB-FISH) (green) used as housekeeping gene and positive control for the RNA FISH assay. (**F**,**G**, and Supplementary Video S1) RNA FISH shows the nuclear localization of lnc-KDM5D-4:1 (red). Each probe was tested with (B,D,F,G, and Supplementary Video S1) or without (**A**,**C**,**E**) addition of 1% of acetic acid (AA) at the cell fixation step. This resulted in no observable nuclear signal for lnc-KDM5D-4:1 when AA is not added to the fixation solution (**E**). (**H**) Real-time PCR lnc-KDM5D-4:1 transcript expression results from nuclear and cytoplasmic RNAs isolated separately from HepG2 cells before reverse transcription, then real-time PCR. Results show a nuclear expression, exclusively, of lnc-KDM5D-4:1 in comparison to ACTB which is approximately half nuclear and half cytoplasmic. Microscopy confocal images represent three independent experiments (*n* = 3). Confocal microscope magnification: 60x.

To gain fundamental insights into the biology and potential cellular role of lnc-KDM5D-4 in the regulation of cellular functions^24^, we examined its subcellular localization using RNA fluorescence *in situ* hybridization (RNA FISH) with single-molecule sensitivity. To achieve a controlled system as background image, the assay was performed without addition of glacial acetic acid (AA) to the fixation solution (Fig. 4A,C,E). This way, although significant cytoplasmic signals were observed for the actin beta (ACTB) housekeeping gene probe (green) (Fig. 4C), any signal obtained was the result of the lnc-KDM5D-4:1 probe (red) (Fig. 4E). These results suggested that lnc-KDM5D-4:1 transcripts were localised only in the nucleus. By adding 1% of AA into the fixation solution, the fluorescence of single-RNA molecule of lnc-KDM5D-4:1 (red) displays a strictly nuclear distribution (Fig. 4F,G and Supplementary Video S1). The ACTB RNA FISH results (Fig. 4C,D) confirmed the efficiency of reagents and methods used for this assay. As negative controls, wells with only secondary probes were tested and no signal displays were seen, as expected, (Fig. 4A,B) confirming that signals obtained for ACTB and lnc-KDM5D-4:1 are not due to the mere expression of the secondary probes. RNA FISH results were then confirmed by real-time PCR using nuclear and cytoplasmic RNAs, separately isolated from HepG2 cells, as shown in Fig. 4H.

### The effects of lnc-KDM5D-4 knockdown on genes in HepG2 cells

**Figure 5:**
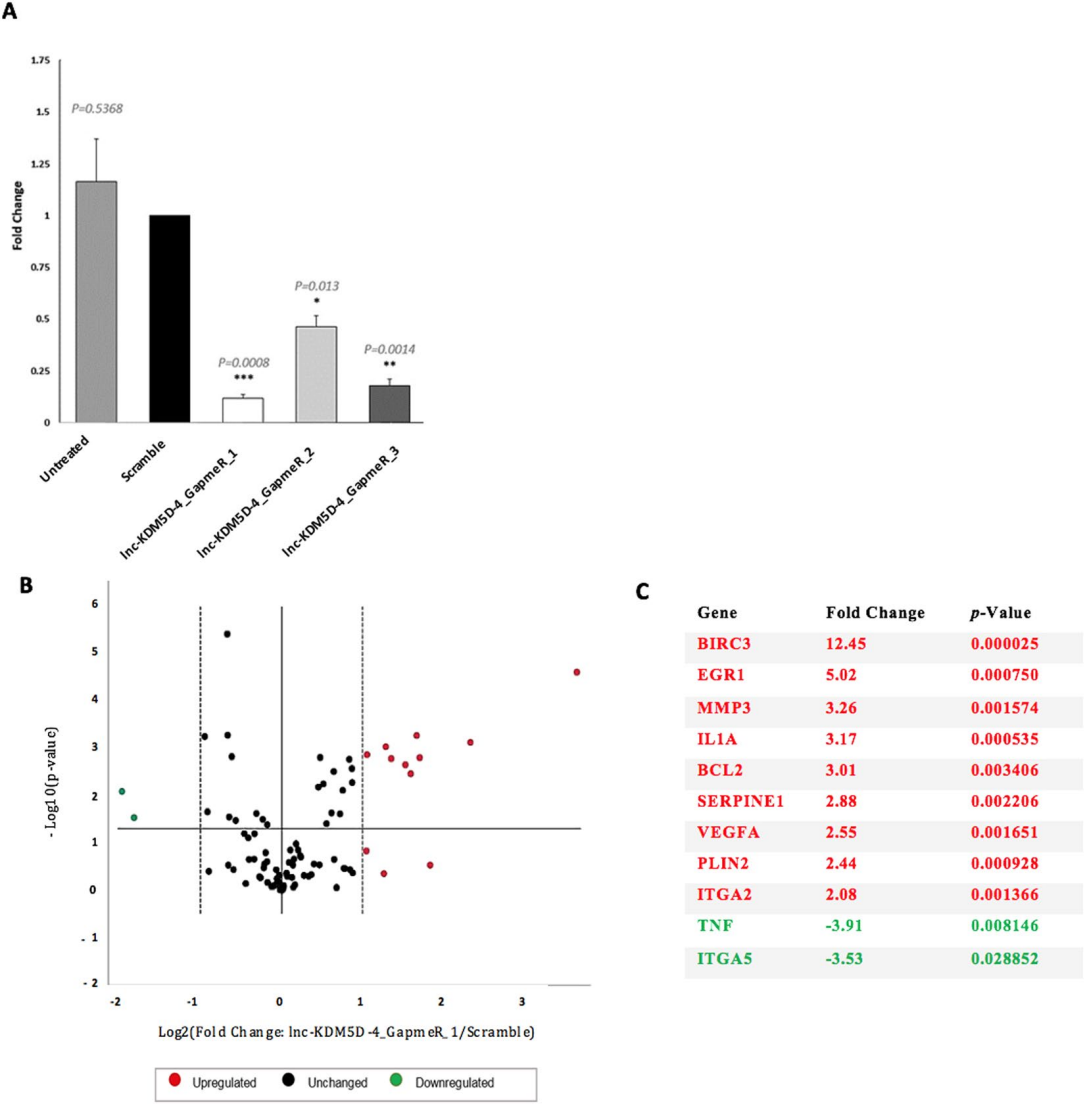
Lnc-KDM5D-4 knockdown and associated atherosclerosis-relevant genes. Cells were transfected with Locked Nucleic Acid (LNA) GapmeRs targeting lnc-KDM5D-4:1 RNA or random scrambled sequence. (**A**) Cells were harvested 48 h after transfection. AO-mediated silencing of lnc-KDM5D-4 was assessed in real-time PCR (normalised to ACTB as housekeeping control gene). The cells demonstrate antisense oligonucleotide knockdown of nuclear-located lnc-KDM5D-4 with the 3-different tested GapmeRs; a highest knockdown efficiency was achieved with the lnc-KDM5D-4_GapmeR_1 [transcript reduction by 88% (Fold change = 0.12; *p-*value = 0.0008)]. Comparison of transfected cells with GapmeRs, and Scramble to Untreated samples (negative control) showed that there was no significant effect of transfection reagent or transfection reagent plus GapmeR respectively on the cell. (**B**,**C**) A Human Atherosclerosis RT^2^ Profiler™ PCR Array was utilized to profile the expression of 84 genes involved in atherosclerosis between HepG2 cells transfected with GapmeR targeting lnc-KDM5D-4 (lnc-KDM5D-4_GapmeR_1) and scrambled cells. Relative expression levels of these genes were normalised to a set of housekeeping genes (Supplementary Table S6). Three independent biological samples were used (*n* = 3). The volcano plot combines a *p*-value statistical test with the fold change enabling identification of genes with both large and small expression changes that are statistically significant are represented; upregulated genes (red), downregulated genes (green), and unchanged genes (black) (**B**). In transfected cells compared to untransfected cells, results determine that 9 genes were significantly over-expressed ((*p-*value < 0.05; Fold Change >2): *BIRC3* (baculoviral IAP repeat containing 3), *EGR1* (early growth response 1), *MMP3* (matrix metallopeptidase 3), *IL1A* (interleukin 1 alpha), *BCL2* (BCL2, apoptosis regulator), *SERPINE1* (serpin family E member 1), *VEGFA* (vascular endothelial growth factor A), *PLIN2* (perilipin 2), and *ITGA2* (integrin subunit alpha 2); and 2 genes were significantly under-expressed ((*p*-value < 0.05; Fold Change <−2.00): *ITGA5* (integrin subunit alpha 5), and *TNF* (tumor necrosis factor) (**C**). The significant dysregulated genes are summarized in Supplementary Figure S2 with their pathways associated with the most represented ‘anti-apoptosis pathway’. Statistical significance: *p-value < 0.05; **p-value < 0.01; ***p-value < 0.001.

To elucidate the potential biological role of Y-linked lnc-KDM5D-4 in the liver, a knockdown using the advanced Locked Nucleic Acid GapmeRs (antisense oligonucleotides (AOs)) that blocked lnc-KDM5D-4:1 activity was performed. This was followed by a gene expression analysis using the RT^2^ Profiler PCR Array. Specifically, we transfected HepG2 cells with GapmeRs targeting lnc-KDM5D-4:1 using three different probes separately (lnc-KDM5D-4_GapmeR_1, lnc-KDM5D-4_GapmeR_2 and lnc-KDM5D-4_GapmeR_3) to target three different sites of lnc-KDM5D-4:1. A random scrambled sequence that contains no known homology to the human, mouse, or rat genome was used as a negative control. Cells without transfection (Untreated) were also used to verify the Scramble (negative control probe) for non-specificity. Cells were harvested 48 h after transfection. AO-mediated silencing of lnc-KDM5D-4 was assessed in real-time PCR. The highest knockdown efficiency was obtained when the cells are treated with 10 nmol/L of the lnc-KDM5D-4_GapmeR_1 with a transcript reduction of 88% (Fold change = 0.12; *p*-value = 0.0008). The comparison of transfected cells with GapmeRs and Scramble (control GapmeR) to Untreated samples showed that there was no significant effect of transfection reagent or transfection reagent plus GapmeR respectively, on the cell (Fig. 5A).

Following that, the Human Atherosclerosis RT^2^ Profiler PCR Array was used to compare cells transfected with the lnc-KDM5D-4_GapmeR_1 and cells transfected with the control GapmeR (Scramble) (Fig. 5B,C). This array profiled the expression of 84 genes related to atherosclerosis. Our results showed that reduced lnc-KDM5D4:1 transcript level in HepG2 cells was associated with changes in expression of 11 genes: *BIRC3* (baculoviral IAP repeat containing 3), *EGR1* (early growth response 1), *MMP3* (matrix metallopeptidase 3), *IL1A* (interleukin 1 alpha), *BCL2* (BCL2, apoptosis regulator), *SERPINE1* (serpin family E member 1), *VEGFA* (vascular endothelial growth factor A), *PLIN2* (perilipin 2), *ITGA2* (integrin subunit alpha 2), *ITGA5* (integrin subunit alpha 5), and *TNF* (tumor necrosis factor) (Fig. 5C). Most of these genes mapped to anti-apoptosis pathway (Supplementary Fig. S2). Finally, the new human tissue-specific network webserver GIANT^22^ was used to highlight the potential functional interactions of these genes with other protein-coding genes in hepatocytes (Supplementary Fig. S3).

**Figure 6:**
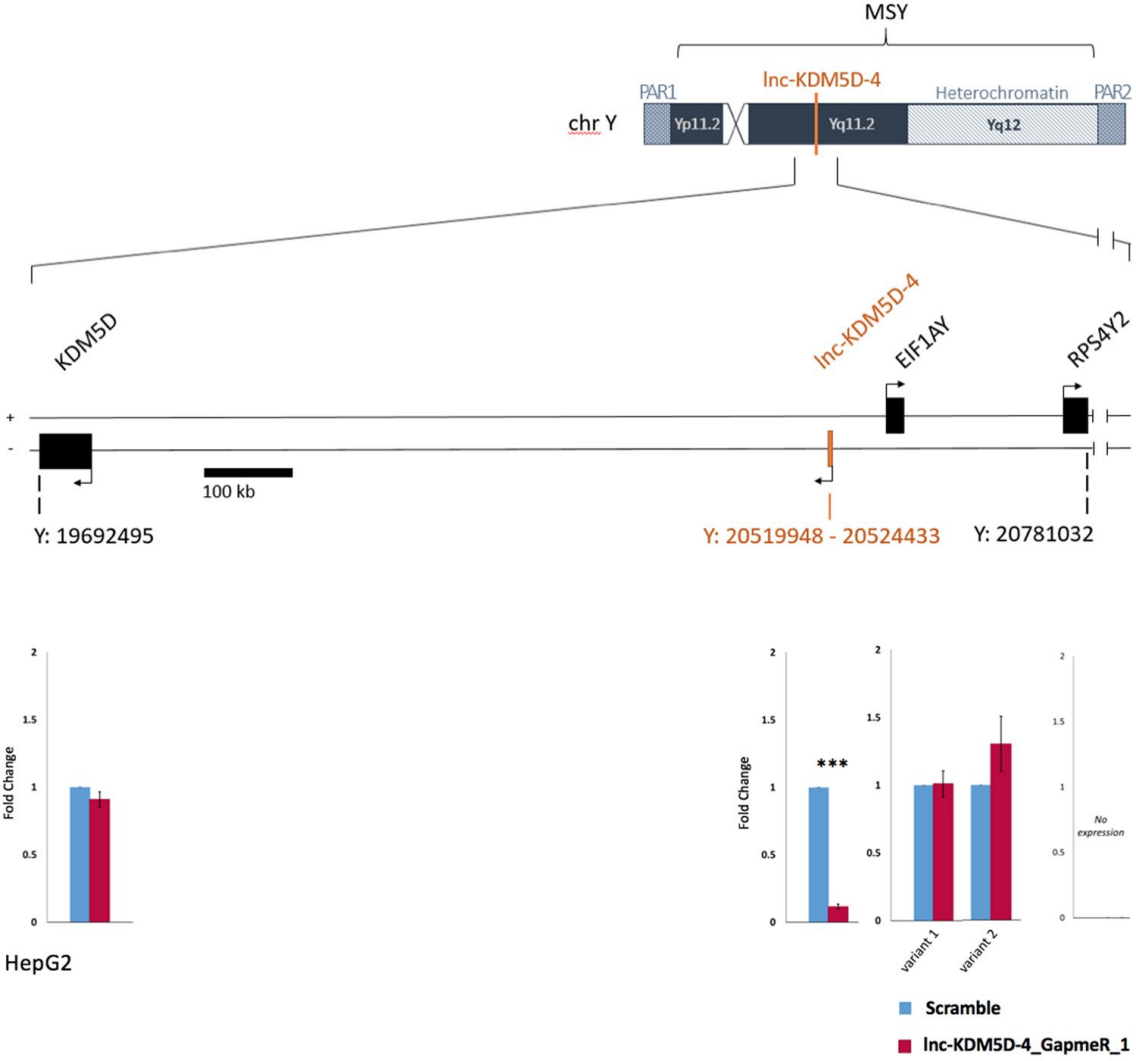
Schematic representation of protein-coding genes surrounding the lncRNA gene lnc-KDM5D-4 on the human Y chromosome. Protein-coding genes within 1.1 MB window centred on lnc-KDM5D-4. Upstream genes: *EIF1AY* (eukaryotic translation initiation factor 1A, Y-linked), which encodes for two transcripts, and *RPS4Y2* (ribosomal protein S4, Y-linked 2). Downstream genes: *KDM5D* (lysine demethylase 5D). The knockdown of lnc-KDM5D-4 in HepG2 cells does not influence the expression of its nearest neighbouring protein-coding genes. MSY: male-specific region of the Y chromosome. PAR1: pseudo-autosomal region 1. PAR2: pseudo-autosomal region 2. Position on the Y chromosome in base pair (bp); Human GRCh38/hg38.

Previous studies on transcriptional regulation models showed that lncRNAs, notably lincRNAs, may operate in *cis* by regulating their immediate neighbouring protein-coding genes leading to either an increase or reduction of their expression^16,25,26^. After confirmation of the lnc-KDM5D-4 knockdown in HepG2 cells, we examined the potential transcriptional activity of the nearest neighbouring protein-coding genes by lnc-KDM5D-4 such as *KDM5D* (lysine demethylase 5D)*, EIF1AY* (eukaryotic translation initiation factor 1A, Y-linked), and *RPS4Y2* (ribosomal protein S4, Y-linked 2) located within 1.1 MB window centred on lnc-KDMM5D-4. Real-time PCR analysis showed that there were no significant changes (*p-*value > 0.05) in the expression of these neighbouring Y chromosome protein-coding genes in cells transfected with the lnc-KDM5D-4_GapmeR_1 compared to the control cells (Scramble) (Fig. 6).

## Discussion

Genetic variation within the Y chromosome has been linked to human lipid levels and cardiovascular disease^2–4,6,27,28^. Here we provide novel data suggestive of a potential role for one of the Y chromosome lincRNA, lnc-KDM5D-4, in the hepatic metabolism of lipids and atherosclerosis. Our findings demonstrate that lnc-KDM5D-4 is a nuclear-retained lincRNA that has a regulatory effect on the mRNA expression of genes such as Perilipin 2 (*PLIN2*), that in turn, lead to increased lipid droplets formation in hepatocytes. We propose that this link may, in part, contribute to the association between certain Y chromosome haplogroups and atherosclerosis.

There is currently no information regarding Y chromosome-linked lncRNAs in human traits and disease. This is due to the fact that the Y chromosome is routinely excluded from genome analysis studies into the identification and the functions of lncRNAs in mammals^29,30^. Indeed, due to the haploid nature of the Y chromosome, the usual methods of analysis (such as genome-wide association studies (GWAS)) cannot be employed to investigate variations. This study provides the first evidence of the expression of Y-linked lincRNA transcripts such as lnc-KDM5D-4:1, lnc-ZFY-1:1, lnc-ZFY-2:1, lnc-RBMY1B-1:1, lnc-RBMY1B-1:4, lnc-USP9Y-1:4, and lnc-HSFY2-3:6 in human hepatocellular carcinoma (HCC). Further research focusing on these lincRNAs between primary human hepatocytes and HepG2 cells should be done as these lincRNAs may also play a role in HCC, and could be potential biomarkers for the diagnosis of this cancer in men. Our data also provides the first evidence for the up-regulation of the Y-linked lincRNA, known as lnc-KDM5D-4, in response to palmitate treatment in HepG2 cells. However, lnc-KDM5D-4 was not differentially expressed or affected in insulin-resistance HepG2 cells, demonstrating that the results found with the steatosis cell model were independent of those found in the insulin-resistance cells. These results confirmed that the significant changes in expression of lnc-KDM5D-4 after the FFA-palmitate treatment was triggered by the steatosis phenotype, and not by the insulin-resistance phenotype. It was suggested that lnc-KDM5D-4 was then implicated in hepatic steatosis which can occur independently of insulin resistance in the liver. Given the role of FFAs in the pathogenesis of CAD^31–33^, these results suggest that lnc-KDM5D-4 may play a role in the hepatic metabolism of lipids – a process of well-known relevance to atherosclerosis. Whether aberrant expression of this lincRNA plays an insignificant role in this context, or if it is a mere consequence of disease pathology remains an open question. Further research on lnc-KDM5D-4 should be done to study the role of this Y-linked lincRNA in steatosis-associated atherosclerosis.

Lnc-KDM5D-4:1 transcripts were found to be expressed in adipose, bladder, brain, colon, esophagus, heart, kidney, liver, lung, prostate, skeletal muscle, small intestine, spleen, testes, thymus, thyroid, trachea, and leucocytes suggesting that this lincRNA is widely expressed in male tissues. These findings provide a novel expression profile for lnc-KDM5D-4 across human healthy tissues. Based on these data, we believe that this lincRNA may have other molecular and physiological roles in men.

The main known function of lncRNAs to date is regulation of gene expression^34^. Lnc-KDM5D-4:1 RNA FISH results suggested that lnc-KDM5D-4:1 is a lincRNA retained within the nucleus of hepatocytes. Concomitantly, these findings were in agreement with the previous observations showing that lncRNA transcripts were enriched in the cell nucleus in HepG2 cells^16^. This clearly showed that lnc-KDM5D-4 plays a potential role in biological functions taking place within the nucleus^24,35^ such as the establishment and maintenance of nuclear domains^36^, shaping of the 3D organization^35,37^, or acting as enhancer-like RNA by activating its neighbouring genes using a *cis*-mediated mechanism^38,39^. Indeed, an exclusively nuclear localization would argue against putative lncRNAs encoding short peptide sequences as translation occurs in the cytoplasm. However, given that its silencing does not affect the expression of its surrounding protein-coding genes, this may argue that it acts in *trans* rather than in *cis*.

**Figure 7:**
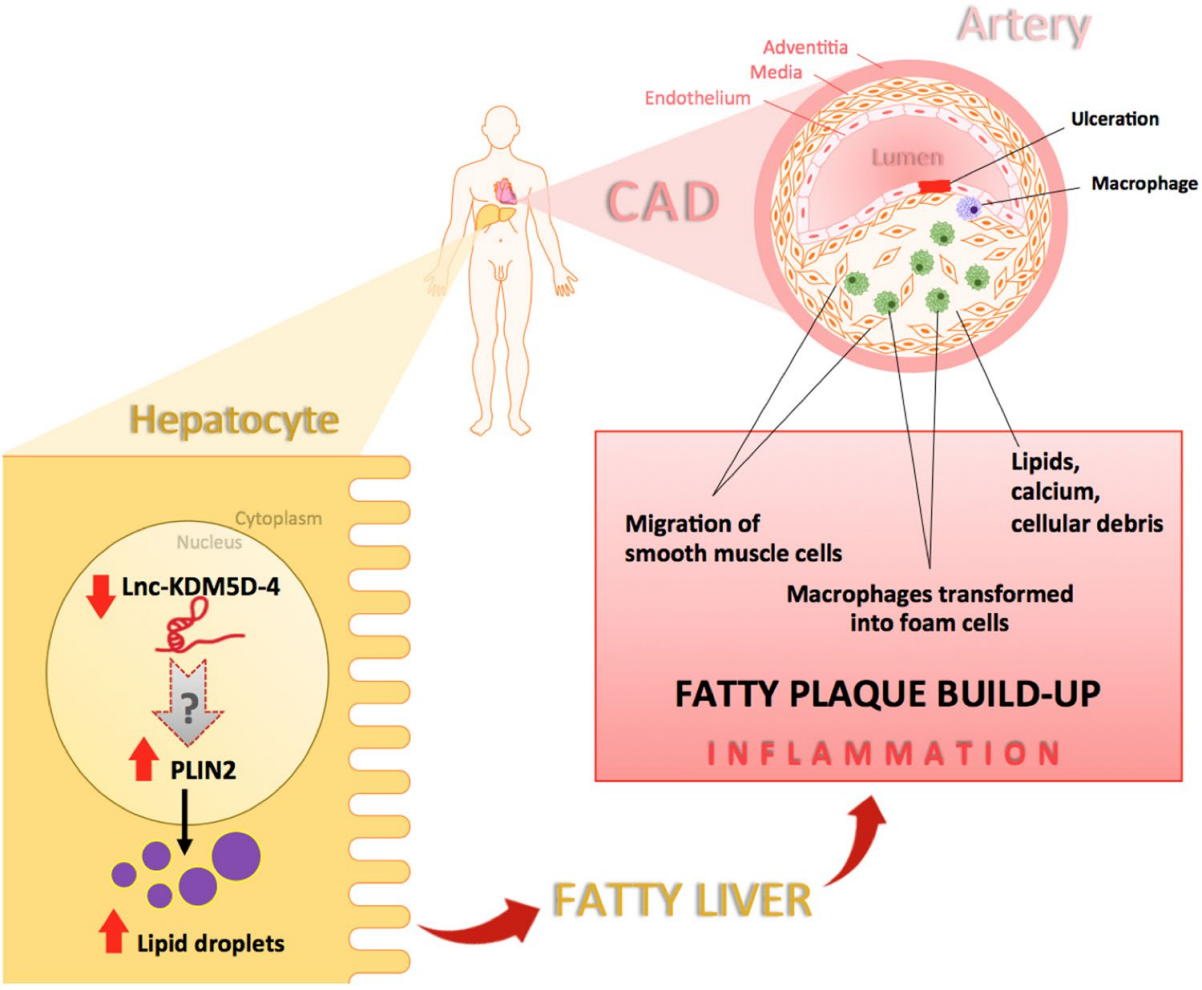
Schematic diagram illustrating the potential role of lnc-KDM5D-4 in atherosclerosis. Atherosclerosis is driven by a chronic inflammatory process. Lipid disturbances and other risk factors are thought to cause endothelial injury resulting in monocyte adhesion and migration to the intima, as well as the release of cytokines and growth factors. Low-density lipoprotein (LDL) particles travelling in the blood and carrying cholesterol and triglycerides from the liver to other body tissues get through the endothelium layer due to their size and density, and become oxidized. After migration to the sub-endothelial space, monocytes differentiate into macrophages, which are then able to ingest oxidized-LDL, forming specialized foam cells. Macrophages are not able to process the oxidized-LDL and ultimately grow and rupture depositing a greater amount of oxidized cholesterol into the artery wall. This triggers the recruitment of more monocytes, thus increasing the inflammation and continuing the cycle. This inflammation leads to subendothelial accumulation of fatty substances called atheromatous plaques. In the hepatocytes, the underexpression of the Y chromosome-linked lincRNA lnc-KDM5D-4 results in an overexpression of the gene perilipin 2 (*PLIN2)* involved in lipid droplet formation within the cells. This increase of expression of *PLIN2* may consequently initiates the ‘fatty liver’ or hepatic steatosis promoting atherosclerosis in the coronary arteries of men.

Among the genes that were affected by lnc-KDM5D-4 knockdown in HepG2 cells, *PLIN2* is known to be implicated in lipid metabolism. This protein-coding gene is located on chromosome 9 and belongs to the perilipin gene family that regulate intracellular lipid storage droplets and very low-density lipoprotein (VLDL) secretion^40^. Overexpression of *PLIN2* has been shown to trigger an increase in lipid droplets formation within hepatocytes via the up-regulation of the peroxisome proliferator-activated receptor gamma isoform (PPAR gamma)^41^. Furthermore, the up-regulation of *PLIN2* was previously associated with liver steatosis^42^ and atherogenesis^43^. More recently, *PLIN2* expression was also associated with atherosclerosis in patients with carotid stenosis^44^. On the other hand, the loss of *PLIN2* has resulted in reduction of liver steatosis and inflammation^45^. An overview of the involvement of lnc-KDM5D-4 and its potential interaction with *PLIN2* in the context of atherosclerosis and CAD is proposed in Fig. 7.

Lnc-KDM5D-4 seems to be not conserved between human and rodents^18^ which could be due to the unique forces that drive the evolution of the Y chromosome. It is therefore not possible to study further this lncRNA in rodents.

In conclusion, this is the first study on lincRNAs on the human Y chromosome and gene expression analysis. We provide evidence for the potential involvement of one of these lncRNAs, lnc-KDM5D-4, in atherosclerosis and CAD, possibly through the lipid metabolism-associated gene *PLIN2* in hepatocytes. Overall, our data adds to the evidence that the human Y chromosome plays an important role in cardiovascular disease in a male specific manner and provides novel insight into potential new therapeutic targets for CAD.

## Electronic supplementary material

Supplementary Information
