# Supplementary material for: A Novel Y-Specific Long Non-Coding RNA Associated with Cellular Lipid Accumulation in HepG2 cells and Atherosclerosis-related Genes
Source: Sci Rep. 2017 Dec 1;7:16710. doi: 10.1038/s41598-017-17165-9 (PMC5711902; doi:10.1038/s41598-017-17165-9)
Supplement: Supplementary file 1 — Supplementary Information [file 41598_2017_17165_MOESM1_ESM.doc]

**A Novel Y-Specific Long Non-Coding RNA Associated with Cellular Lipid Accumulation in HepG2 cells and Atherosclerosis-related Genes**

Elsa Molina^1^, Guat S. Chew^1^, Stephen A. Myers^2^, Elyse M. Clarence^1^,
James M. Eales^3^, Maciej Tomaszewski^3^, ^*^Fadi J. Charchar^1,4,5^

**SUPPLEMENTARY INFORMATION**

**Detailed Materials and Methods**

**Primer design**

*LNCipedia* database was used because the sequences of the lincRNAs selected were available exclusively through this database as of January 2013. Housekeeping genes (HKGs), actin beta *(ACTB*) and ubiquitin C *(UBC)* were selected in accordance to the tissue type, following literature recommendations^1-3^. Primer sequences for the Y-linked protein-coding genes and HKGs studied are available in Supplementary Table 2. All the primers were ordered through Integrated DNA Technologies® website.

**HepG2 cell culture and treatments**

The cell culture medium was replaced twice per week and HepG2 cells were subcultured following the American Type Culture Collection (ATCC)’s recommendations. [Note: HepG2 cells used for all the following experiments were between passage 7 and passage 10]. Cells were seeded into cell culture plates, 6-well, at a density of ~2x10^5^/mL and allowed to grow until the cells reached ~70% confluence before treatments. After overnight FBS-free medium culturing, cells were treated with FFA palmitate (Palmitic acid powder, Sigma®) and/or with insulin (Recombinant insulin solution, Sigma®) as described by Lee *et al.* ^4^. The palmitate was dissolved in chloroform (vehicle) as recommended by the manufacturer’s instructions. The palmitate and insulin solutions were then freshly diluted in DMEM 10% FBS to a final concentration of 0.3mM and 100nM, respectively. Cells were treated over either a 24 or 48-hour time course in triplicate wells with the palmitate and/or insulin solution to develop a steatosis model and/or an insulin-resistance cell model. Control cells were treated with either the vehicle or with DMEM 10% FBS only.

**Oil Red O cell staining**

To confirm steatosis induced by palmitate in HepG2 cells, cellular lipid droplets were stained using Oil Red O (ORO), according to Hepatic Lipid Accumulation/Steatosis Assay (abcam®). Before treatment, cells were seeded into 96-well plates at a density of 10^4^ cells/well as recommended by the manufacturer and incubated overnight in FBS-free medium as described by ^4^. The next day, cells were treated with chloroquine (25µmol/L) as positive control, a compound known to induce steatosis (provided by the assay) for 3 days as recommended by the manufacturer or with palmitate (0.3mmol/L) and/or vehicle (chloroform, as recommended by the manufacturer) for 24h in sextuplicate wells. At the end of this incubation, cells were fixed, then stained with the ORO solution. Lipid accumulation was assessed by two methods; quantification of lipid accumulation by adding the Dye Extraction Solution (provided by the kit) and measuring the absorbance at 520nm, and intracellular distribution of lipid droplets by examination of the cells through a light microscope after adding hematoxylin solution as a nuclear stain.

**Protein extraction and Western Blot analysis**

Total cellular protein from HepG2 cells treated with insulin over 48h was isolated by scraping the cells with Pierce® RIPA buffer (supplemented with Halt protease and phosphatase inhibitor cocktail; Thermo Fisher Scientific®) and then the samples were placed on ice for 1h with constant vortexing every 10 min. The protein samples were centrifuged at 13,000 rpm for 10 min and the supernatant was collected. Total protein concentration was measured using a BCA kit (BIORAD®) as outlined by the manufacturer’s instructions. Total soluble protein (50μg) was resolved on a 4-15% SDS-PAGE gradient gel (BIORAD®) and transferred to a nitrocellulose membrane. The membranes were blocked overnight in 5% skim milk in TBS-Tween 20 followed by an overnight incubation with protein kinase B (Akt) (or insulin receptor (IR)) (Cell Signalling 1:5000 Catalogue No:9272), or phosphorylated AKT (pAkt) (or pIR) (Cell Signalling, 1:5000, Catalogue No: 4058) antibodies. Following 4 x 15 minute washes, the membrane was incubated with anti-Rabbit HRP (Cell Signalling, Catalogue No:7074) 1:5000 for 1h at room temperature. Immunoreactive signals were detected using enhanced chemiluminescence SuperSignal West Pico Substrate (Pierce®) and visualized by autoradiography or a UVITEC Alliance digital imaging system (Thermo Fisher Scientific®). [Note: to assess protein loading consistency, the membranes were stripped with Restore Plus Western Blot Stripping Buffer (Thermo Fisher Scientific®) by incubating the membrane in the buffer for 15 min at room temperature. Membranes were subsequently washed in TBS- Tween and blocked with 5% skim milk before adding the primary antibody].

**Synthesis of cDNA and real-time PCR**

RNA (2µg) was reverse transcribed into cDNA with the High Capacity Reverse Transcription Kit (Life Technologies®). Real-time PCR was assessed in triplicate and carried out using SYBR® Green (Bioline®) according to the manufacturer’s instructions. The level of gene expression was measured using Applied Biosystems® ViiA™ 7 Real-Time PCR system (Life Technologies®). Specifically, 20ng (2µL) of cDNA was added to 2.5µL of SYBR® Green real-time PCR reaction mix (Bioline®), 200nmol/L of each of the forward and reverse primer (Integrated DNA Technologies®), and 0.3µL of nuclease free water in a final volume of 5µL. Real-time PCR was then performed on the ViiA7 quantitative PCR machine using the following method: PCR reaction protocol consisted of 1 cycle at 95°C for 2 min, followed by 40 cycles of denaturation at 95°C for 5 sec, annealing at 60°C for 10 sec, and an extension at 72°C for 20 sec. This was followed by the melt curve analysis which was carried out at 95°C for 15 sec, 60°C for 1 min and 95°C for 15 sec. CT results were normalised with two housekeeping genes, ACTB (or GAPDH) and UBC, and the same significant results were obtained with both. [Note: only results from ACTB or GAPDH are represented here].

**RNA fluorescence *in situ* hybridization (RNA FISH)**

RNA FISH with single-molecule sensitivity was performed using QuantiGene® ViewRNA ISH Cell Assay reagents (Affymetrix®). This is based on the advanced branched DNA technology ^5^ which is performed with four distinct probe sets: a gene-specific probe set composed of ten or more oligonucleotide probe pairs that are complementary to the target sequence; a pre-amplifier probe that hybridizes to gene-specific probes; multiple amplifier probes that hybridize to the pre-amplifier probe; and labelled probes that attach to the amplifier probes. The resulting construct yields a bright concentrated fluorescence. For this experiment, a custom probe set specific for the human lnc-KDM5D-4:1 transcript, was designed and synthesized by Affymetrix as “TYPE 1” (1452 nucleotides in length targeting all the 2 exons; product ID: VA1-16549-01). A probe set specific for human ACTB housekeeping mRNA (product ID: VA4-10293-01, “Type 4”) was used as a cytoplasmic control to assist in interpreting spatial localisation of lnc-KDM5D-4:1 RNA transcripts. Firstly, HepG2 cells (between 1.5x10^5^ and 2x10^5^/mL) were cultured under complete cell culture medium on poly-L-lysine (0.01%) (Sigma®) coated eight-chambered dishes (Lab-Tek®) overnight according to the manufacturer’s instructions to reach 70-90% confluency at the beginning of the assay. The next day, cells were fixed in paraformaldehyde 4% in Phosphate-buffered saline (PBS) (ChemCruz™) for 30 min at room temperature. The permeabilisation procedure was then amended and glacial acetic acid (AA) was added to the fixation solution to detect nuclear RNAs. For that, we followed Affymetrix specialists’ advice about the permeabilisation procedure of the cells during the assay and we also did some literature searching on RNA FISH and nuclear targets. Three different concentrations of AA (0.5%, 1% and 2%) were tested as recommended by ^5^. From these results, 1% AA concentration was determined to be the optimal concentration for use on hepatocytes. This concentration was also determined by ^5^ to be the most optimal with use on another human cell model. An increased amount of false positive signals for lnc-KDM5D-4:1 with 2% of AA were observed. In contrast, weak signals were observed with 0.5% AA for the same probe [data not shown]. The permeabilisation procedure was amended and glacial AA (1%) was added, then the RNA FISH assay from Four-Chambered Dish Format protocol was performed according to the manufacturer’s instructions. At the end of the procedure, after staining nuclei with the 4',6-diamidino-2-phenylindole (DAPI), coverslips were mounted in ProLong® Gold antifade reagent (Life Technologies®) to preserve a better fluorescent signal, then sealed with black nail polish. Images were taken using a Nikon® Eclipse Ti confocal microscope (Nikon®).

**Reverse transcription and Atherosclerosis RT^2^ Profiler™ PCR Array**

The Human Atherosclerosis RT^2^ Profiler PCR Array (PAHS-038ZE) was purchased from SA Biosciences (Qiagen®; Catalogue No. 330231). This array profiles the expression of 84 genes related to atherosclerosis (Supplementary Tables 3 & 4). cDNA synthesis using the RT^2^ First Strand Kit was performed as described by the manufacturer (Qiagen®). Potential genomic DNA was eliminated from 400ng of total RNA using Buffer GE at 42°C for 5 min, and the reaction was stopped at 4°C for at least 1 min. The RNA was then reverse transcribed in a total of 20µL reaction volume for 15 min at 42°C, then the reaction was stopped by heating the sample at 95°C for 5 min. The real-time PCR for the RT^2^ atherosclerosis array was performed as outlined in the guidelines supplied by the manufacturers (Qiagen®). cDNA (102µL) from the RT^2^ First Strand Kit was added to 650µL of 2 x RT^2^ SYBR Green and 548µL of RNase-free water. In each well of the atherosclerosis array 384-well plate, 10µL of sample was added. A real-time PCR was performed on the Applied Biosystems ViiA™ 7 Real-Time PCR system (Life Technologies®) using the following cycling conditions: 1 cycle at 95°C for 10 min followed by 40 cycles of amplification at 95°C for 15 sec and 95°C for 1 min. This was followed by a melt curve analysis which was carried out at 95°C for 15 sec, 60°C for 1 min and 95°C for 15 sec. Data analysis from 3 independent experiments (3 biological samples per groups; Supplementary Table 5) using the delta delta C_T_ method was performed using the Web portal [www.SABiosciences.com/pcrarraydataanalysis.php](http://www.SABiosciences.com/pcrarraydataanalysis.php). Finally, the human tissue-specific network webserver GIANT ^6^ (http://giant.princeton.edu/) was used to generate the potential tissue-specific functional interactions between the atherosclerosis-relevant genes.

**Supplemental Figures and Figure Legends**

**Figure S1: Western blot analysis of phosphorylated protein kinase B (A), Total Akt (B), phosphorylated insulin-receptor (C), and Total IR (D) - uncropped gel images**. Antibodies were used to immunodetect the levels of pAkt (60kDa), Total Akt (60kDa), pIR (95kDa) and Total IR (95kDa) in an increasingly insulin-resistance HepG2 cell line. For protein phosphorylation detection, 10nM insulin was added for 30 min before cell lysates harvest at the indicated time. Western blot analysis was performed on two independent experiments.

**A B**

**
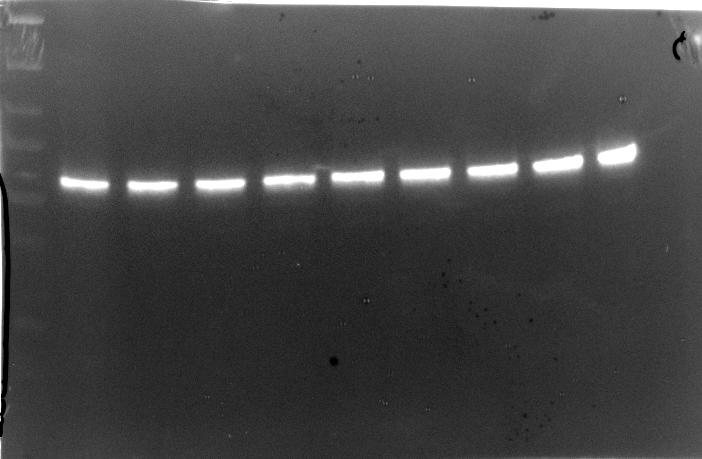

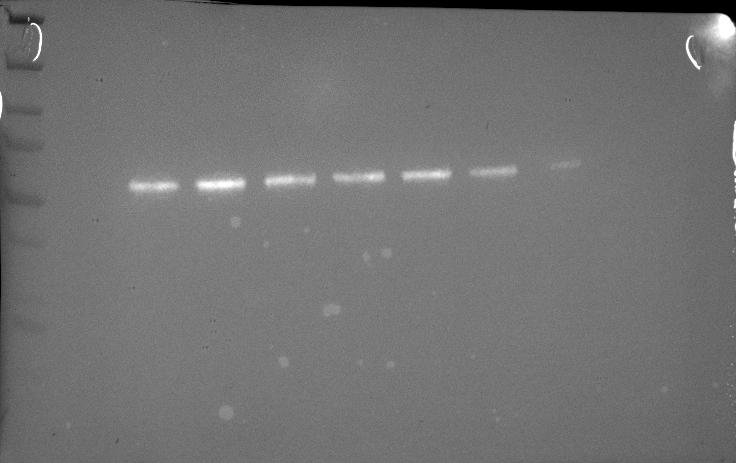
**

100kDa

75kDa

50kDa

**C D**

**
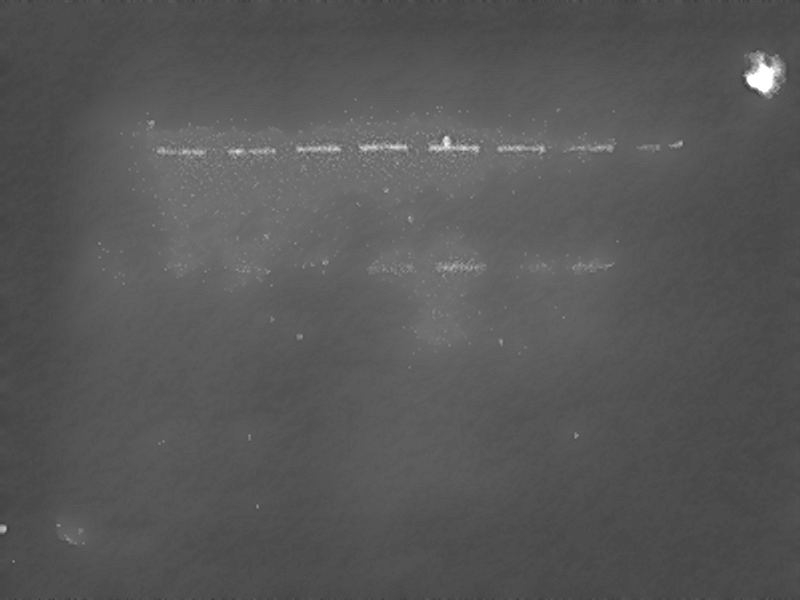

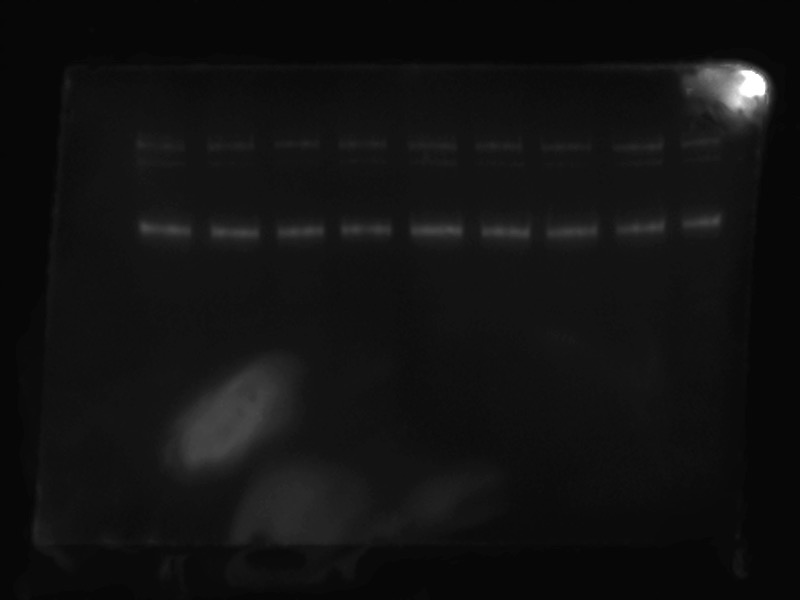
**

**Figure S2: Atherosclerosis PCR Array significant dysregulated genes and associated pathways.** A Human Atherosclerosis RT2 Profiler™ PCR Array was utilised to profile the expression of 84 genes involved in the regulation and enzymatic pathways of atherosclerosis between HepG2 cells transfected with GapmeR targeting lnc-KDM5D-4 and scrambled cells. The significant dysregulated genes are summarized here with their pathways associated. Relative levels of these genes were normalized to a set of housekeeping genes (Supplementary Table 6). Statistical significance: * p<0.05, ** p<0.01, *** p<0.001.


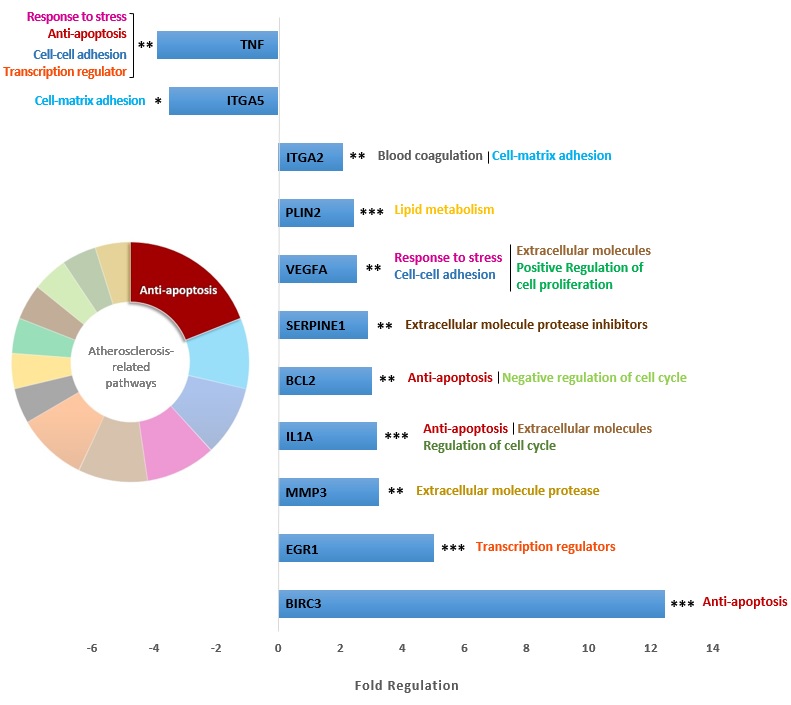


**Figure S3: Interaction network of genes differentially expressed after lnc-KDM5D-4 knockdown in the hepatocyte transcriptome.** Network data is derived from the GIANT (*Genome-scale Integrated Analysis of gene Networks in Tissues)* webserver. Each node is a different gene, which is sized by the number of connections in the network, differentially expressed genes are colored red. Edges represent interaction strength and are colored from weak (turquoise) to strong interaction (dark blue) between gene partners, interaction strength is a combined score determined by co-expression and co-occurrence in known biological pathways. GIANT data was extracted with a minimum relationship confidence of 0.8.

**
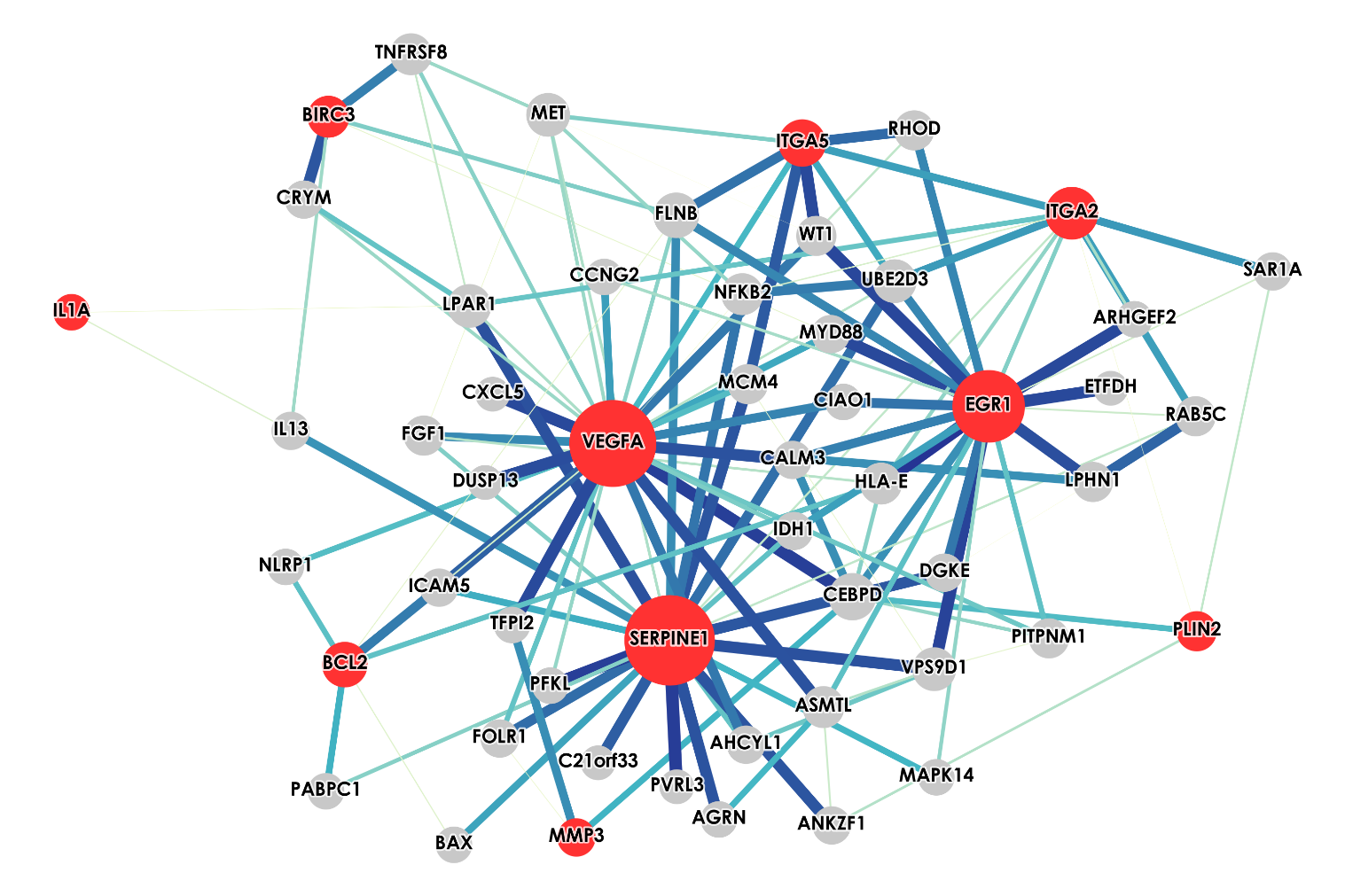
**

**Supplemental Tables**

**Table S1: Real-time primer sequences for lincRNAs annotated on LNCipedia.** All the primers have been designed to reach an optimal melting temperature (Tm) of 60°C and a product size between 70 and 200 base pair (bp).

| **LNCipedia**  **gene ID** | **LNCipedia**  **Transcript ID** | **Sequence 5’-3’** | **Tm**  **(°C)** | **Product length (bp)** |
| --- | --- | --- | --- | --- |
| **lnc-KDM5D-4** | lnc-KDM5D-4:1 | For: AAGTTGGGTCACACAGTCCC | 59.82 | 139 |
|  |  | Rev: GGGACTACCAAGTATGGGCA | 58.79 |  |
| **lnc-ZFY-1** | lnc-ZFY-1:1 | For: ACAACCACTCAAGGTCTGCAA | 60.06 | 109 |
|  |  | Rev: TGGCAGTGTAAGTATGGCACA | 59.65 |  |
|  | lnc-ZFY-1:3 | For: ACAACCACTCAAGGTCTGCAA | 60.06 | 138 |
|  |  | Rev: GAGCGCCACTGGGAATCAAG | 61.37 |  |
| **lnc-ZFY-2** | lnc-ZFY-2:1 | For: AACTGGCTGAGCTTTCCAAGA | 59.86 | 132 |
|  |  | Rev: CTCAGACATCAACAGCAATCATCA | 59.37 |  |
| **lnc-RBMY1B-1** | lnc-RBMY1B-1:1 | For: AGTCTCTTGCAACACCACCT | 59.26 | 118 |
|  |  | Rev: AAACTGGCACATGAGCCTCC | 60.55 |  |
|  | lnc-RBMY1B-1:4 | For: TCTCTTGCAACACCACCTGAG | 60.20 | 117 |
|  |  | Rev: TAAACTGGCACATGAGCCTCC | 60.34 |  |
| **lnc-RBMY1J-1** | lnc-RBMY1J-1:1 | For: GAATCCTCGGGAGAGTCCCA | 60.40 | 82 |
|  |  | Rev: GGCTCTGCTTGAGGCTTCTTA | 60.07 |  |
|  | lnc-RBMY1J-1:2 | For: CAGGACCTTCACGTCACTCC | 60.04 | 106 |
|  |  | Rev: CCCCTCACCAGCTTGTATCC | 59.82 |  |
|  | lnc-RBMY1J-1:3 | For: AAGACCACACCACTCCCAAG | 59.53 | 127 |
|  |  | Rev: GGCTGGGAACTGAATTTGAGC | 59.80 |  |
| **lnc-USP9Y-1** | lnc-USP9Y-1:4 | For: TGGTTTATGGTCATTGAACCTTGT | 58.86 | 171 |
|  |  | Rev: CCAGCTCTGTGGACAAAACG | 59.41 |  |
| **lnc-HSFY2-3** | lnc-HSFY2-3:6 | For: CAGGCATGTGCAGGAGGTTT | 60.90 | 163 |
|  |  | Rev: AGTTGCAGGCAGAGTGATGAA | 59.93 |  |

**Table S2: Primer sequences for PubMed annotated genes.** Primers have been designed to reach an optimal melting temperature (Tm) of 60°C and a product size between 70 and 200 base pair (bp).

| **Gene name** | **Gene symbol** | **Sequence 5’-3’** | **Tm**  **(°C)** | **Product length (bp)** |
| --- | --- | --- | --- | --- |
| ***Actin, beta*** | **ACTB** | For: CGCGAGAAGATGACCCAGAT | 59.98 | 119 |
|  |  | Rev: GAGTCCATCACGATGCCAGT | 60.02 |  |
| ***Eukaryotic translation initiation factor 1A, Y-linked, variant 1*** | **EIF1AY** | For: TGGACGATTGGAAGCATTGTG | 59.19 | 126 |
|  |  | Rev: TCCTGATAGTCCCGTAGACCA | 59.15 |  |
| ***Eukaryotic translation initiation factor 1A, Y-linked, variant 2*** | **EIF1AY** | For: GAAGAGGTCTCACGAGGCTG | 59.83 | 84 |
|  |  | Rev: TCATTTTTACCCCTGCGCCT | 59.96 |  |
| ***Hepatocellular carcinoma up-regulated long non-coding RNA*** | **HULC** | For: AAACTCTGAAGTAAAGGCCGGA | 59.63 | 74 |
|  |  | Rev: CTTGCTTGATGCTTTGGTCTGT | 59.70 |  |
| ***Lysine (K)-specific demethylase 5D*** | **KDM5D** | For: CCACAGCATCCCCCTTAGAC | 59.82 | 262 |
|  |  | Rev: GCCCACCTCCACTTTGCTCAT | 59.96 |  |
| ***ribosomal protein S4, Y-linked 2*** | **RPS4Y2** | For: TTCCCGCTGTTTATCACCGC | 61.02 | 171 |
|  |  | Rev: TCGATGGACGAGGTGCAAAT | 59.75 |  |
| ***Ubiquitin C*** | **UBC** | For: TCAGACGAAGGGCGCAGC | 60.74 | 83 |
|  |  | Rev: AAGGCCGAGTCTTATGAGCAG | 59.86 |  |

**Table S3: Functional Gene Grouping – RT^2^ Profiler™ PCR Array Human Atherosclerosis (Qiagen®).**(<https://www.sabiosciences.com/rt_pcr_product/HTML/PAHS-038Z.html>)


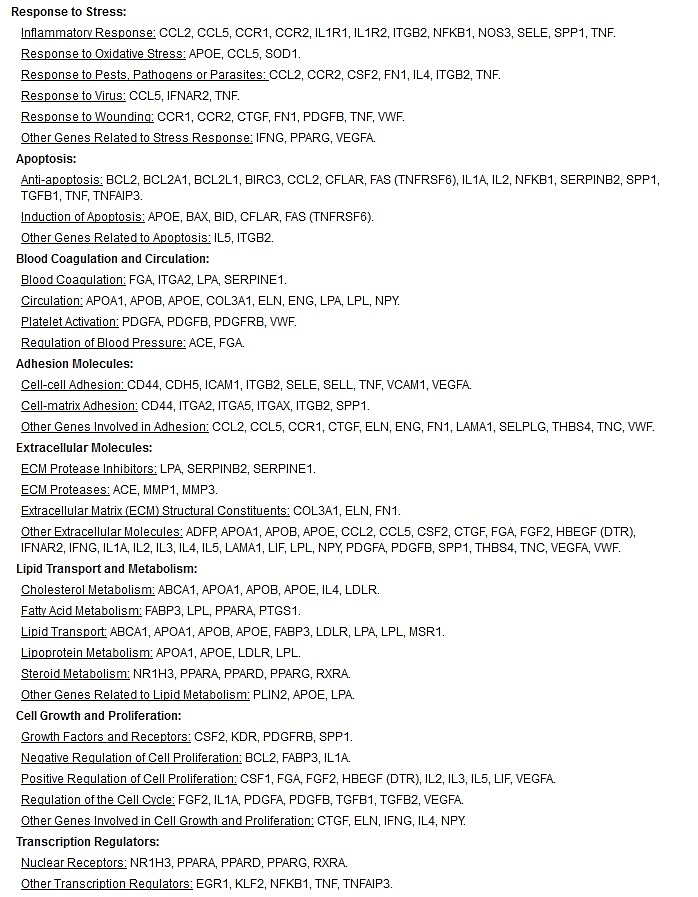


**Table S4: Gene table – RT^2^ Profiler™ PCR Array Human Atherosclerosis.**

| **Position** | **RefSeq Number** | **Symbol** | **Description** |
| --- | --- | --- | --- |
| A01 | NM_005502 | ABCA1 | ATP-binding cassette, sub-family A (ABC1), member 1 |
| A02 | NM_000789 | ACE | Angiotensin I converting enzyme (peptidyl-dipeptidase A) 1 |
| A03 | NM_000039 | APOA1 | Apolipoprotein A-I |
| A04 | NM_000384 | APOB | Apolipoprotein B (including Ag(x) antigen) |
| A05 | NM_000041 | APOE | Apolipoprotein E |
| A06 | NM_004324 | BAX | BCL2-associated X protein |
| A07 | NM_000633 | BCL2 | B-cell CLL/lymphoma 2 |
| A08 | NM_004049 | BCL2A1 | BCL2-related protein A1 |
| A09 | NM_138578 | BCL2L1 | BCL2-like 1 |
| A10 | NM_001196 | BID | BH3 interacting domain death agonist |
| A11 | NM_001165 | BIRC3 | Baculoviral IAP repeat containing 3 |
| A12 | NM_002982 | CCL2 | Chemokine (C-C motif) ligand 2 |
| B01 | NM_002985 | CCL5 | Chemokine (C-C motif) ligand 5 |
| B02 | NM_001295 | CCR1 | Chemokine (C-C motif) receptor 1 |
| B03 | NM_001123396 | CCR2 | Chemokine (C-C motif) receptor 2 |
| B04 | NM_000610 | CD44 | CD44 molecule (Indian blood group) |
| B05 | NM_001795 | CDH5 | Cadherin 5, type 2 (vascular endothelium) |
| B06 | NM_003879 | CFLAR | CASP8 and FADD-like apoptosis regulator |
| B07 | NM_000090 | COL3A1 | Collagen, type III, alpha 1 |
| B08 | NM_000757 | CSF1 | Colony stimulating factor 1 (macrophage) |
| B09 | NM_000758 | CSF2 | Colony stimulating factor 2 (granulocyte-macrophage) |
| B10 | NM_001901 | CTGF | Connective tissue growth factor |
| B11 | NM_001964 | EGR1 | Early growth response 1 |
| B12 | NM_000501 | ELN | Elastin |
| C01 | NM_000118 | ENG | Endoglin |
| C02 | NM_004102 | FABP3 | Fatty acid binding protein 3, muscle and heart (mammary-derived growth inhibitor) |
| C03 | NM_000043 | FAS | Fas (TNF receptor superfamily, member 6) |
| C04 | NM_000508 | FGA | Fibrinogen alpha chain |
| C05 | NM_002006 | FGF2 | Fibroblast growth factor 2 (basic) |
| C06 | NM_002026 | FN1 | Fibronectin 1 |
| C07 | NM_001945 | HBEGF | Heparin-binding EGF-like growth factor |
| C08 | NM_000201 | ICAM1 | Intercellular adhesion molecule 1 |
| C09 | NM_000874 | IFNAR2 | Interferon (alpha, beta and omega) receptor 2 |
| C10 | NM_000619 | IFNG | Interferon, gamma |
| C11 | NM_000575 | IL1A | Interleukin 1, alpha |
| C12 | NM_000877 | IL1R1 | Interleukin 1 receptor, type I |
| D01 | NM_004633 | IL1R2 | Interleukin 1 receptor, type II |
| D02 | NM_000586 | IL2 | Interleukin 2 |
| D03 | NM_000588 | IL3 | Interleukin 3 (colony-stimulating factor, multiple) |
| D04 | NM_000589 | IL4 | Interleukin 4 |
| D05 | NM_000879 | IL5 | Interleukin 5 (colony-stimulating factor, eosinophil) |
| D06 | NM_002203 | ITGA2 | Integrin, alpha 2 (CD49B, alpha 2 subunit of VLA-2 receptor) |
| D07 | NM_002205 | ITGA5 | Integrin, alpha 5 (fibronectin receptor, alpha polypeptide) |
| D08 | NM_000887 | ITGAX | Integrin, alpha X (complement component 3 receptor 4 subunit) |
| D09 | NM_000211 | ITGB2 | Integrin, beta 2 (complement component 3 receptor 3 and 4 subunit) |
| D10 | NM_002253 | KDR | Kinase insert domain receptor (a type III receptor tyrosine kinase) |

| **Position** | **RefSeq Number** | **Symbol** | **Description** |
| --- | --- | --- | --- |
| D11 | NM_016270 | KLF2 | Kruppel-like factor 2 (lung) |
| D12 | NM_005559 | LAMA1 | Laminin, alpha 1 |
| E01 | NM_000527 | LDLR | Low density lipoprotein receptor |
| E02 | NM_002309 | LIF | Leukemia inhibitory factor (cholinergic differentiation factor) |
| E03 | NM_005577 | LPA | Lipoprotein, Lp(a) |
| E04 | NM_000237 | LPL | Lipoprotein lipase |
| E05 | NM_002421 | MMP1 | Matrix metallopeptidase 1 (interstitial collagenase) |
| E06 | NM_002422 | MMP3 | Matrix metallopeptidase 3 (stromelysin 1, progelatinase) |
| E07 | NM_002445 | MSR1 | Macrophage scavenger receptor 1 |
| E08 | NM_003998 | NFKB1 | Nuclear factor of kappa light polypeptide gene enhancer in B-cells 1 |
| E09 | NM_000603 | NOS3 | Nitric oxide synthase 3 (endothelial cell) |
| E10 | NM_000905 | NPY | Neuropeptide Y |
| E11 | NM_005693 | NR1H3 | Nuclear receptor subfamily 1, group H, member 3 |
| E12 | NM_002607 | PDGFA | Platelet-derived growth factor alpha polypeptide |
| F01 | NM_002608 | PDGFB | Platelet-derived growth factor beta polypeptide |
| F02 | NM_002609 | PDGFRB | Platelet-derived growth factor receptor, beta polypeptide |
| F03 | NM_001122 | PLIN2 | Perilipin 2 |
| F04 | NM_005036 | PPARA | Peroxisome proliferator-activated receptor alpha |
| F05 | NM_006238 | PPARD | Peroxisome proliferator-activated receptor delta |
| F06 | NM_015869 | PPARG | Peroxisome proliferator-activated receptor gamma |
| F07 | NM_000962 | PTGS1 | Prostaglandin-endoperoxide synthase 1 (prostaglandin G/H synthase and cyclooxygenase) |
| F08 | NM_002957 | RXRA | Retinoid X receptor, alpha |
| F09 | NM_000450 | SELE | Selectin E |
| F10 | NM_000655 | SELL | Selectin L |
| F11 | NM_003006 | SELPLG | Selectin P ligand |
| F12 | NM_002575 | SERPINB2 | Serpin peptidase inhibitor, clade B (ovalbumin), member 2 |
| G01 | NM_000602 | SERPINE1 | Serpin peptidase inhibitor, clade E (nexin, plasminogen activator inhibitor type 1), member 1 |
| G02 | NM_000454 | SOD1 | Superoxide dismutase 1, soluble |
| G03 | NM_000582 | SPP1 | Secreted phosphoprotein 1 |
| G04 | NM_000660 | TGFB1 | Transforming growth factor, beta 1 |
| G05 | NM_003238 | TGFB2 | Transforming growth factor, beta 2 |
| G06 | NM_003248 | THBS4 | Thrombospondin 4 |
| G07 | NM_002160 | TNC | Tenascin C |
| G08 | NM_000594 | TNF | Tumor necrosis factor |
| G09 | NM_006290 | TNFAIP3 | Tumor necrosis factor, alpha-induced protein 3 |
| G10 | NM_001078 | VCAM1 | Vascular cell adhesion molecule 1 |
| G11 | NM_003376 | VEGFA | Vascular endothelial growth factor A |
| G12 | NM_000552 | VWF | Von Willebrand factor |
| H01 | NM_001101 | ACTB | Actin, beta |
| H02 | NM_004048 | B2M | Beta-2-microglobulin |
| H03 | NM_002046 | GAPDH | Glyceraldehyde-3-phosphate dehydrogenase |
| H04 | NM_000194 | HPRT1 | Hypoxanthine phosphoribosyltransferase 1 |
| H05 | NM_001002 | RPLP0 | Ribosomal protein, large, P0 |
| H06 | SA_00105 | HGDC | Human Genomic DNA Contamination |
| H07 | SA_00104 | RTC | Reverse Transcription Control |
| H08 | SA_00104 | RTC | Reverse Transcription Control |
| H09 | SA_00104 | RTC | Reverse Transcription Control |
| H10 | SA_00103 | PPC | Positive PCR Control |
| H11 | SA_00103 | PPC | Positive PCR Control |
| H12 | SA_00103 | PPC | Positive PCR Control |

**Table S5: Data analysis and data quality control (QC) – RT^2^ Profiler™ PCR Array Human Atherosclerosis.**

**
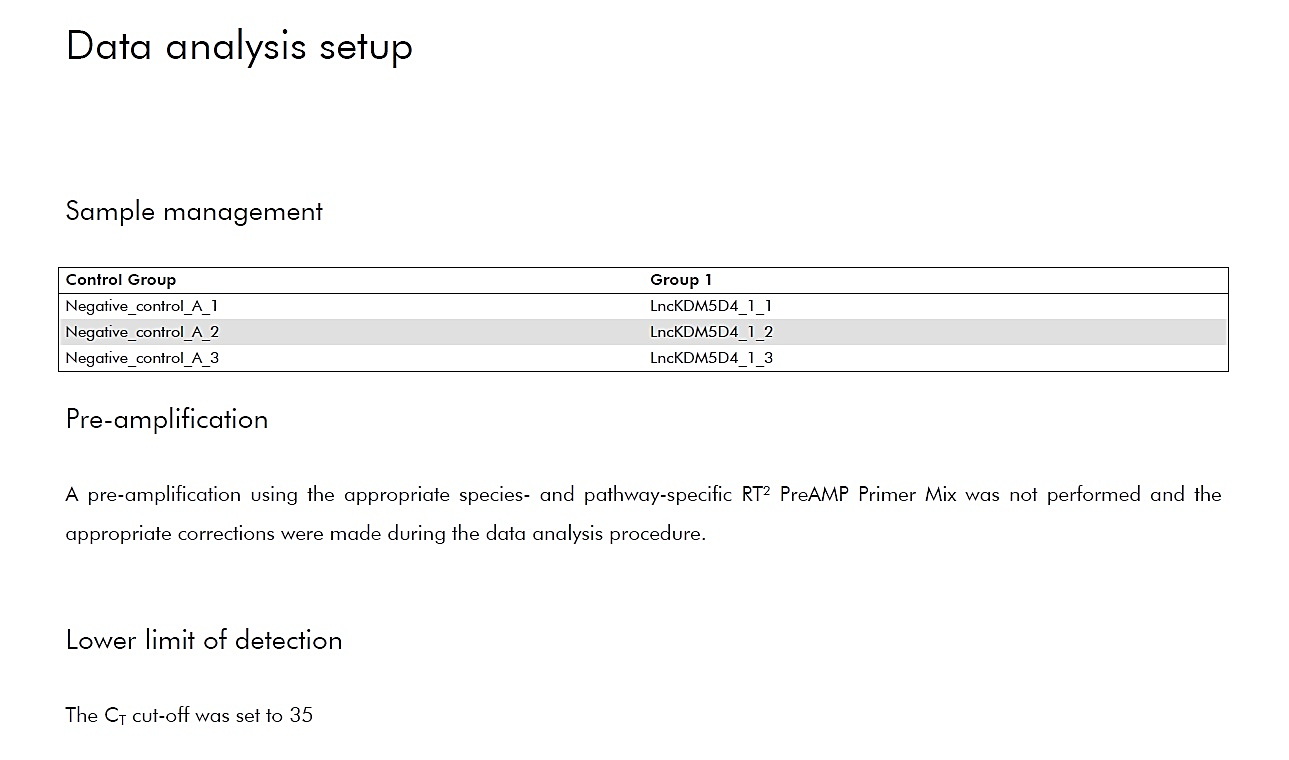
**


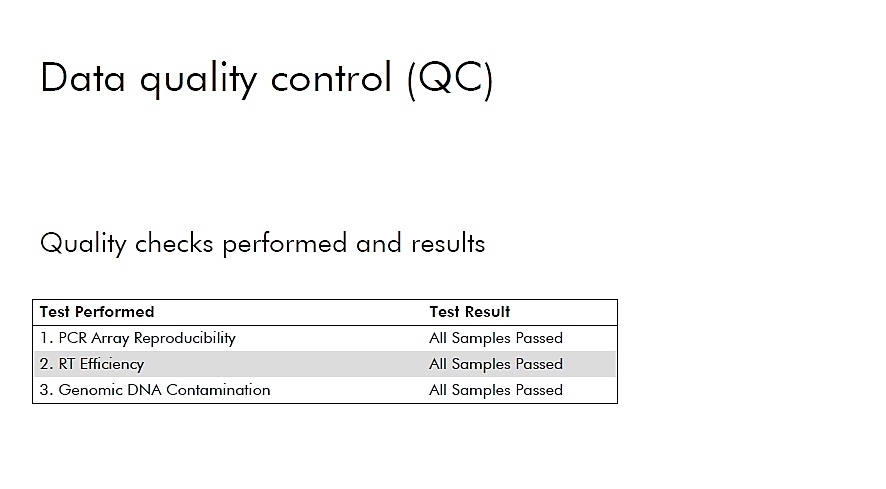


**Table S6: Housekeeping genes used for the normalization analysis – RT^2^ Profiler™ PCR Array Human Atherosclerosis.**


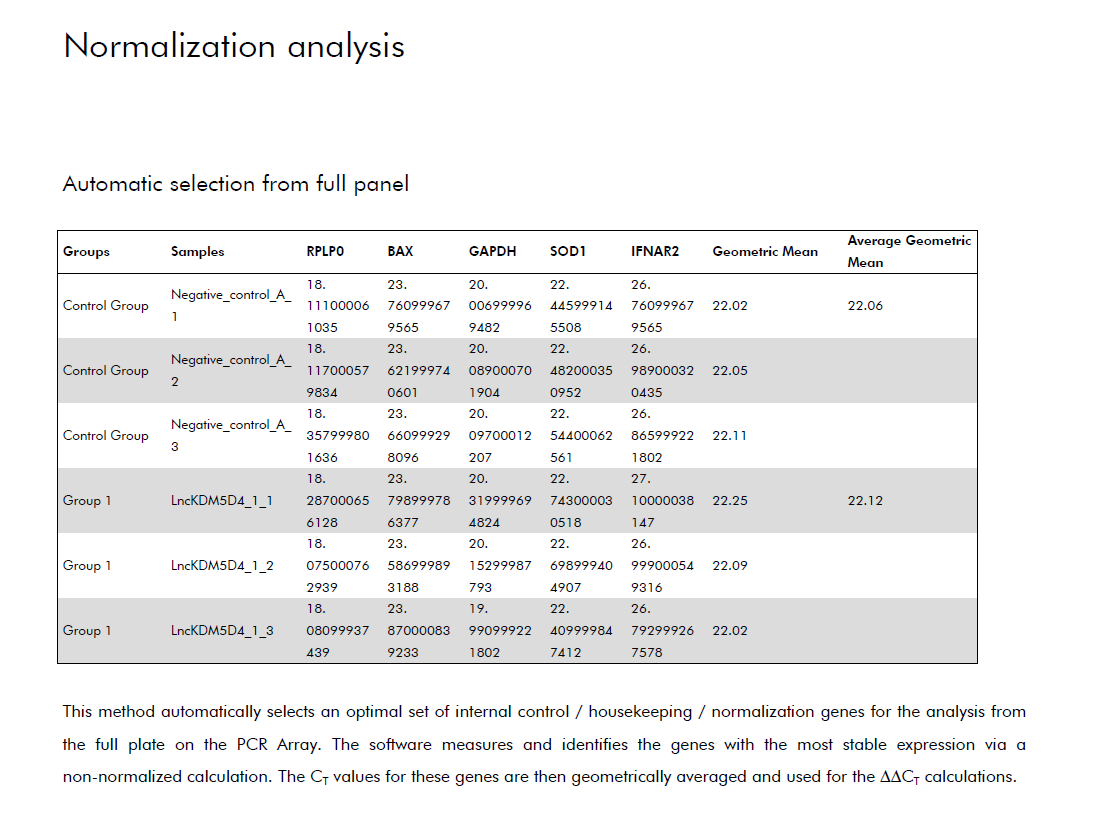


**Supplemental References**

**1 Dheda, K. *et al.* The implications of using an inappropriate reference gene for real-time reverse transcription PCR data normalization. *Anal Biochem* 344, 141-143, doi:10.1016/j.ab.2005.05.022 (2005).**

**2 Ledderose, C., Heyn, J., Limbeck, E. & Kreth, S. Selection of reliable reference genes for quantitative real-time PCR in human T cells and neutrophils. *BMC Res Notes* 4, 427, doi:10.1186/1756-0500-4-427 (2011).**

**3 Rebouças, E. d. L. *et al.* Real time PCR and importance of housekeepings genes for normalization and quantification of mRNA expression in different tissues. *Brazilian Archives of Biology and Technology* 56, 143-154 (2013).**

**4 Lee, J. Y., Cho, H. K. & Kwon, Y. H. Palmitate induces insulin resistance without significant intracellular triglyceride accumulation in HepG2 cells. *Metabolism* 59, 927-934, doi:10.1016/j.metabol.2009.10.012 (2010).**

**5 Battich, N., Stoeger, T. & Pelkmans, L. Image-based transcriptomics in thousands of single human cells at single-molecule resolution. *Nat Methods* 10, 1127-1133, doi:10.1038/nmeth.2657 (**2013**).**

**6 Greene, C. S. *et al.* Understanding multicellular function and disease with human tissue-specific networks. *Nat Genet* 47, 569-576, doi:10.1038/ng.3259 (2015).**
